# Subtle adversarial image manipulations influence both human and machine perception

**DOI:** 10.1038/s41467-023-40499-0

**Published:** 2023-08-15

**Authors:** Vijay Veerabadran, Josh Goldman, Shreya Shankar, Brian Cheung, Nicolas Papernot, Alexey Kurakin, Ian Goodfellow, Jonathon Shlens, Jascha Sohl-Dickstein, Michael C. Mozer, Gamaleldin F. Elsayed

**Affiliations:** 1 Google, Mountain View, CA USA; 2 Google DeepMind, Mountain View, CA USA; 3 Present Address: Department of Cognitive Science, University of California, San Diego, CA USA; 4 Present Address: University of California, Berkeley, CA USA; 5 Present Address: MIT Brain and Cognitive Sciences, Cambridge, MA USA

**Keywords:** Human behaviour, Visual system, Computer science

## Abstract

Although artificial neural networks (ANNs) were inspired by the brain, ANNs exhibit a brittleness not generally observed in human perception. One shortcoming of ANNs is their susceptibility to adversarial perturbations—subtle modulations of natural images that result in changes to classification decisions, such as confidently mislabelling an image of an elephant, initially classified correctly, as a clock. In contrast, a human observer might well dismiss the perturbations as an innocuous imaging artifact. This phenomenon may point to a fundamental difference between human and machine perception, but it drives one to ask whether human sensitivity to adversarial perturbations might be revealed with appropriate behavioral measures. Here, we find that adversarial perturbations that fool ANNs similarly bias human choice. We further show that the effect is more likely driven by higher-order statistics of natural images to which both humans and ANNs are sensitive, rather than by the detailed architecture of the ANN.

## Abstract (web summary)

Artificial neural networks (ANNs) are vulnerable to subtle adversarial perturbations that yield misclassification errors. Here, behavioral studies demonstrate that adversarial perturbations that fool ANNs similarly bias human choice.

## Introduction

Artificial neural networks (ANNs) have produced revolutionary advances in machine intelligence, from image recognition^1^ to natural language understanding^2^ to robotics^3^. The inspiration for ANNs was provided by biological neural networks (BNNs)^4^. For instance, convolutional ANNs adopt key characteristics of the primate visual system, including its hierarchical organization, local spatial connectivity, and approximate translation equivariance^5,6^. The historical relationship between ANNs and BNNs has also led to ANNs being considered as a framework for understanding biological information processing. Visual representations in ANNs are strikingly similar to neural activation patterns in primate neocortex^7–12^, and ANNs have been successful in accounting for a range of behavioral phenomena in human perception, learning, and memory^13–19^.

However, qualitative differences exist between human and machine perception. Architecturally, human perception has capacity limitations generally avoided in machine vision systems, such as bottlenecks due to spatial attention and the drop off in visual acuity with retinal eccentricity^20^. In terms of training environments, human perception is immersed in a rich multi-sensory, dynamical, three-dimensional experience, whereas standard training sets for ANNs consist of static images curated by human photographers^20^. While these differences in architecture, environment, and learning procedures seem stark, they may not reflect differences in underlying knowledge and capacities, but instead constraints in manifesting the knowledge (i.e., the performance-competence distinction raised by Firestone^21^). Nonetheless, these differences may have behavioral consequences. ANNs are found to be brittle relative to human perception in handling various forms of image corruption^22^. One possible explanation for this finding is that machine perception is heavily influenced by texture whereas human perception is guided by shape^23^. The robustness gap between machine and human perception is narrowing as ANNs and training data set increase in scale, reaching the point where machines surpass human robustness on some forms of input noise^24^. Nonetheless, even as the robustness gap narrows, humans make systematically different classification errors than machines^24^.

**Fig. 1:**
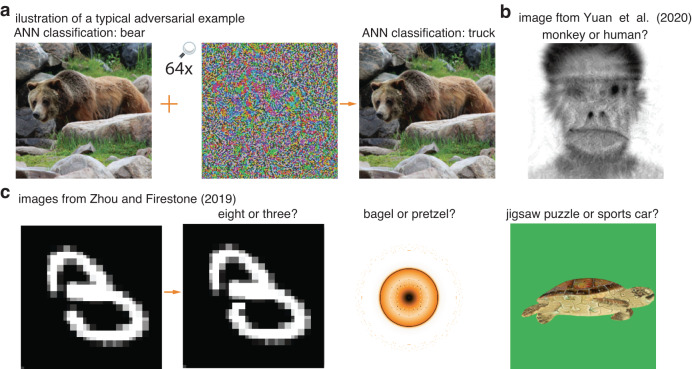
Examples of adversarial images used as stimuli in past research. **a** A subtle perturbation added to a bear image that causes an ANN to switch its classification decision from bear to truck, similar to those first demonstrated in Figure 5 of Szegedy et al., 2013^25^. Original image shown here was obtained from MS-COCO dataset^62^. In this and subsequent figures, perturbations are scaled up for better visualization. **b** An adversarial attack that causes face-selective neurons in the macaque inferotemporal cortex to predict a perturbed human face image as monkey not human, obtained with permission from Yuan et al. 2020^35^. **c** Various adversarial images used in human behavioral studies by Zhou and Firestone, 2019^33^. This paper presents studies that go beyond the work in (**b**) and (**c**) by using perturbations more closely resembling those illustrated in Figure 5 of Szegedy et al.^25^, which seem relatively subtle and innocuous, the key properties of adversarial examples that made them `intriguing' in earlier work^25^. Illustration images in panel (**c**) were obtained with permission from Papernot et al.^70^, Nguyen et al.^71^, and Athalye et al.^72^, left to right respectively.

One particular class of ANN errors has attracted significant interest in the machine-learning community because the errors seem incongruous with human perception. These errors are produced by *adversarial image perturbations*, subtle image-specific modulations of individual pixels that are designed to alter ANN categorization to different coarse image classes^25–27^, as illustrated in Fig. 1a. This adversarial effect often transfers to ANN models trained on a different data set^25^, with a different algorithm^28^, or even to machine learning algorithms with fundamentally different architectures^25^ (e.g., adversarial examples designed to fool a convolution neural network may also fool a decision tree). What makes these perturbations so remarkable is that to casual human observers, the image category is unchanged and the adversarial perturbations are interpreted—to the extent they are even noticed—as irrelevant noise.

The standard procedure for generating adversarial perturbations starts with a pretrained ANN classifier that maps RGB images to a probability distribution over a fixed set of classes^25^. When presented with an uncorrupted image, the ANN will typically assign a high probability to the correct class. Any change to the image, such as increasing the red intensity of a particular pixel, will yield a slight change to the output probability distribution. Adversarial images are obtained by searching—via gradient descent—for a perturbation of the original image that causes the ANN to reduce the probability assigned to the correct class (an untargeted attack) or to assign a high probability to some specified alternative class (a targeted attack). To ensure that the perturbations do not wander too far from the original image, an *L*_*∞*_-norm constraint is often applied in the adversarial machine-learning literature; this constraint specifies that no pixel can deviate from its original value by more than ±*ϵ*, with *ϵ* usually much smaller than the [0–255] pixel intensity range^27^. The constraint applies to pixels in each of the RGB color planes. Although this restriction does not prevent individuals from detecting changes to the image, with an appropriate choice of *ϵ* the predominant signal indicating the original image class in the perturbed images is mostly intact. Yet ANNs largely change their predictions in response to adversarial perturbations.

These errors seem to point to a troubling fragility of ANNs, which makes them behave in a manner that is counter intuitive and ostensibly different than human perception, suggesting fundamental flaws in their design. Ilyas et al.^29^ propose that the existence of adversarial examples is due to ANNs exploiting features that are predictive but not causal, and perhaps ANNs are far more sensitive to these features than humans. Kim et al.^30^ further argued that neural mechanisms in the human visual pathway may filter out the signal contained in adversarial perturbations. However, Ford et al.^31^ make a theoretical claim that any classifier, human or machine, will be susceptible to adversarial examples in high dimensional input spaces if the classifier achieves less than perfect generalization to more standard types of corruption (e.g., Gaussian noise) or to naturally occurring variation in the input. As humans sometimes make classification mistakes, it may be inevitable that they also suffer from adversarial examples. However, it is not inevitable that ANNs and BNNs are susceptible to the same adversarial examples.

Although the fascination with adversarial perturbations stems from the assumption that ANNs are fooled by a manipulation to which humans are believed to be impervious, some evidence has arisen contradicting this assumption. Han et al.^32^ used fMRI to probe neural representations of adversarial perturbations and found that the early visual cortex represents adversarial perturbations differently than Gaussian noise. This difference is a necessary condition for human behavioral sensitivity to adversarial perturbations. Several research teams have shown that primate forced-choice classification decisions of modulated or synthetic images can be predicted by the responses of ANNs. First, Zhou and Firestone^33^ performed a series of experiments on a variety of fooling images (Fig. 1c) that ranged from synthetic shapes and textures to images having perceptually salient modulations. Although human-ANN agreement is found, Dujmović et al.^34^ argue that the agreement is weak and dependent on the choice of adversarial images and the design of the experiment. Second, Yuan et al.^35^ trained an ANN to match the responses of face-selective neurons in the macaque inferotemporal cortex and then used this model to modulate images toward a target category (Fig. 1b). Both human participants and monkey subjects showed the predicted sensitivity to the modulations. In each of these two experiments, the image modulations were not subtle and a human observer could make an educated guess about how another observer would respond in a forced-choice judgment. As such, it remains an open question whether people are influenced by images that possess the intriguing property^25^ that first drew machine-learning researchers to adversarial examples—that they are corruptions to natural images that are relatively subtle and might easily be perceived as innocuous noise.

In this work, we investigate whether adversarial perturbations—a subordinate signal in the image—influence individuals in the same way as they influence ANNs. We are not exploring here whether predominant and subordinate signals may have separation in human cognition, but rather that both signals may influence human perception. The challenge in making this assessment is that under ordinary viewing conditions, individuals are so strongly driven by the predominant signal that categorization responses ignore the subordinate signal. In contrast, ANNs clearly have a different balance of influence from the predominant and subordinate signals such that the subordinate signal dominates the decision of ANNs. Setting aside this notable and well-appreciated difference, which is a key reason for the interest in adversarial attacks, the question remains whether the subordinate signal reflects some high-order statistical regularity to which both ANNs and BNNs are sensitive. If so, we obtain even more compelling evidence for ANNs as a model of human perception; and if not, we can point to a brittleness of ANNs that should be rectified before trust is placed in them to make perceptual decisions. We conduct behavioral experiments in which participants performed forced-choice classification of a briefly presented perturbed image, or participants inspected pairs of perturbed images with no time constraint and selected the one that better represented an object class. We find converging evidence from these experiments suggesting that subordinate adversarial signals that heavily influence ANNs can also influence humans in the same direction. We further find the ANN properties that underlie this perceptual influence and identify reliance on shape cues as an important characteristic enhancing alignment with human perception.

## Results

### Adversarial perturbations increase human classification errors with brief presentations

**Fig. 2:**
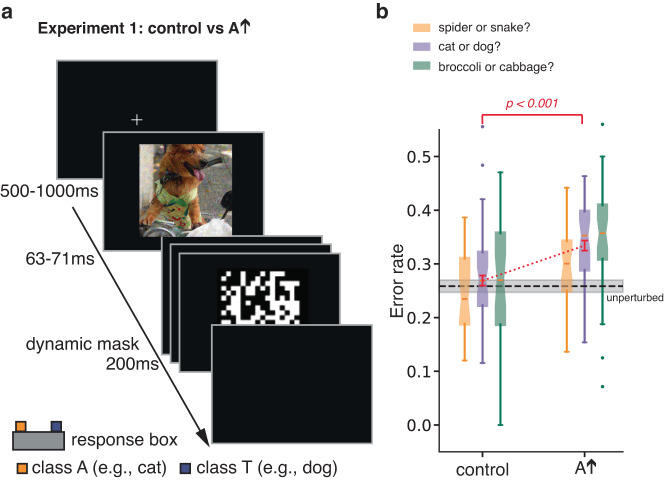
Experiment 1: Adversarial perturbations increase errors when participants are asked to classify briefly presented images. **a** Following fixation, a stimulus image is presented briefly followed by a dynamic, high contrast mask. Participants chose between two classes, the target class *T* (dog in the example) and an adversarial class *A* (cat in the example). Images used in the experiment are obtained from ImageNet dataset^61^, but the image used here as an illustration is obtained from MS-COCO dataset^62^. **b** Box-and-whisker plots show the human error-rate distribution obtained from a pool of *n* = 38 independent participants who performed a max of three discrimination conditions (spider vs. snack *n* = 24, cat vs. dog *n* = 35, broccoli vs. cabbage *n* = 32). We use Tukey conventions: box lower border, middle line, and upper border show 25th percentile, median, and 75th percentile, respectively, and whiskers show the lowest and highest points within 1.5 × the interquartile range. The mean error rate across conditions is reliably higher for adversarial versus control stimuli (*t*(91) = 4.463, *p* < 0.001, Cohen’s *d* = 0.66, 95% CI of difference between means = [0.04, 0.09], one-tailed *t*-test). Red error bars indicate ± 1 standard error of the mean (SE). The black dashed line is the baseline error rate (±1 SE) for unperturbed images.

In an initial experiment, we examined human classification responses to brief, masked presentations of adversarial images. By restricting exposure time to increase classification errors, the experiment aimed to increase individuals’ sensitivity to aspects of the stimuli that might otherwise not have influenced a classification decision. We created adversarial perturbations to images of a true class *T* by optimizing the perturbation such that an ensemble of ANNs (prepended with an artificial retinal blurring layer, see Supplementary Note 1) produces a classification preference for an adversarial class, *A*. We refer to the perturbed image as *A↑*. Participants were asked to make a forced choice between *T* and *A* (Fig. 2a). We also tested participants on control images formed by top-down flipping adversarial perturbations obtained in the *A↑* condition. This simple transformation breaks the pixel-to-pixel correspondence between the adversarial perturbation and the image and largely obliterates the adversarial effect on ANNs while preserving norms and other statistics of the perturbation. Our results show that participants are more likely to choose the adversarial class *A* with *A↑* images than with control images (Fig. 2b).

The increase in error rate appears to demonstrate a consistent influence of adversarial perturbations on both ANNs and BNNs. However, one can raise three concerns with this experiment. First, the specificity of the attack observed in ANNs—where targeting class *A* results in higher probability specifically for class *A*—has not been established by the current experimental paradigm. Forced choice precludes the possibility that participants perceive images to be of a third class. Participants may be sensitive only to the fact that images are less clean examples of class *T* in the *A↑* condition. Second, the perturbation magnitude we used (*ϵ* = 32), is larger than is typical in generating adversarial examples for ANNs. With lower magnitudes, measuring reliable effects on human classification may be challenging. Third, with large perturbations, it is possible that the increased error rate is due to the perturbations obscuring image regions critical to discriminating classes *T* and *A* in the *A↑* condition, but not in the control condition, which lacks the pixel-to-pixel correspondence between perturbation and source image. Indeed, in a time-unlimited variant of the cat versus dog discrimination of Experiment 1 (Experiment SI-5), error rates are slightly higher in the *A↑* condition (0.135 vs. 0.089; *t*(50) = 3.19, *p* < 0.001, Cohen’s *d* = 0.45, 95% CI of difference in means is greater than 0.02, right-tailed), admitting the possibility that some of the effect observed in Experiment 1 is due to obfuscation.

### Adversarial perturbations bias human choice in extended viewing

**Fig. 3:**
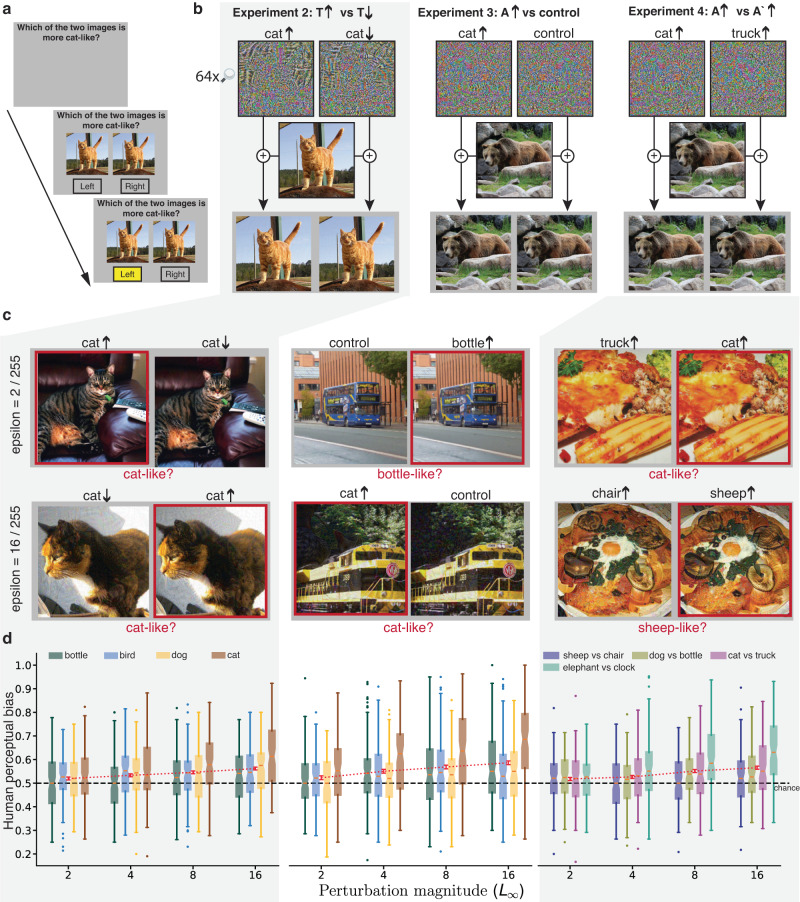
Experiments 2–4: Adversarial examples systematically bias choice. **a** Participants are shown two perturbations of the same image, of true class *T*, and are asked to select the image which is more like an instance of some adversarial class *A*. The image pair remains visible until a choice is made. **b** One of the two choices is an adversarial perturbation that increases the probability of classifying the image as *A*, denoted *A↑*. Experiment 2: *T* = *A*; the second image is perturbed to be less *A*-like, denoted *A↓*. Experiment 3: *T* ≠ *A*; the second image is formed by adding a right-left flipped version of the adversarial perturbation, which controls for the magnitude of the perturbation while removing the image-to-perturbation correspondence. Experiment 4: *T* ≠ *A*; the second image is an adversarial perturbation toward a third class ${A}^{{\prime} }$, denoted ${A}^{{\prime} }\uparrow$. **c** We show examples of adversarial images which empirically yielded human responses consistent with those of the ANN (indicated by the red box) for *ϵ* = 2 and 16, corresponding to the lowest and largest perturbation magnitudes used in these experiments. Example images in (**a**–**c**) are obtained from the Microsoft COCO dataset^62^ and OpenImages dataset^63^; images in (**a**, **b**, and **c**) left are used for illustration outside of our stimulus set due to license limitations. **d** Box plots (same convention as Fig. 2c) quantifying participant bias toward *A↑* (where *A* = *T* for Experiment 2 and *A* ≠ *T* for Experiments 3 and 4), as a function of *ϵ* for four different conditions (each a different adversarial class *A*) collected from n=389 participants for Experiment 2 (cat *n* = 100, dog *n* = 100, bird *n* = 90, bottle *n* = 99), *n* = 396 participants for Experiment 3 (cat *n* = 96, dog *n* = 100, bird *n* = 101, bottle *n* = 99) and *n* = 389 independent participants for Experiment 4 (sheep vs chair *n* = 97, dog vs bottle *n* = 99, cat vs truck *n* = 98, elephant vs clock *n* = 94). The red points (with ± 1 SE bars) indicate the mean across conditions. The black dashed line indicates the performance of a random strategy that is insensitive to the adversarial perturbations.

Experiment 1 used brief, masked presentations to limit the influence of the original-image class (the predominant signal) on responses, and thereby to reveal sensitivity to adversarial perturbations (the subordinate signal). We designed three additional experiments that had the same goal but avoided the need for large-magnitude perturbations and limited-exposure viewing. In these experiments, the predominant signal in an image could not systematically guide response choice, allowing the influence of the subordinate signal to be revealed. In each of these experiments, a pair of unmasked nearly-identical stimuli are presented and remain visible until a response is chosen (Fig. 3a). The pair of stimuli share the same predominant signal, i.e., they are both modulations of the same underlying image, but they have different subordinate signals (Fig. 3b). Participants are asked to select the image that is more like an instance of a target class (Fig. 3c). In Experiment 2, the two stimuli are modulations of an image of class *T*, one perturbed such that ANNs predict it to be more *T*-like and one perturbed to be less *T*-like (*T↑* and *T↓*, respectively). In Experiment 3, the stimuli are modulations of an image belonging to a true class *T*, one perturbed to alter ANN classification toward a target adversarial class *A* (*A↑*) and the other using the same perturbation except flipped right-left as a control condition (control); this control serves to preserve norms and other statistics of the perturbation, but is more conservative than the control in Experiment 1 because left and right sides of an image may have more similar statistics than the upper and lower parts of an image. In Experiment 4, the pair are also modulations of an image of a true class *T*, one perturbed to be more *A*-like and one to be more like a third class, ${A}^{{\prime} }$ (*A↑* and ${A}^{{\prime} }\uparrow$, respectively). Trial blocks alternated between participants being asked to choose the more *A*-like image and the more ${A}^{{\prime} }$-like (See also Supplementary Fig. 1 for analogous experiments using brief and masked presentations).

In each experiment, the ANN has higher confidence in the target class for one stimulus of the pair over the other because of the differential effect of the subordinate signal (Fig. 3b); neither choice conflicts with the true class of the original image. And in each experiment, human perception is consistently biased by the adversarial perturbation in the direction predicted by the ANN (Fig. 3d; E2: $F(1,385)=156.7,p < .001,{\eta }_{p}^{2}=0.29$, 95% CI of perceptual bias = [0.03, 0.05], E3: $F(1,392)=140.6,p < .001,{\eta }_{p}^{2}=0.26$, 95% CI of perceptual bias = [0.05, 0.07], E4: $F(1,385)=126.7,p < .001,{\eta }_{p}^{2}=0.25$, 95% CI of perceptual bias = [0.05, 0.07]; see also Supplementary Table 9 for a nonparametric Wilcoxin test of significance). Human perceptual bias is robust across target classes and grows with *ϵ*. Conducting a two factor ANOVA, we observe a main effect for the target class, indicating that participants are more sensitive to some classes than others (E2: *F*(3, 385) = 12.94, *p* < 0.001, ${\eta }_{p}^{2}=0.09$, E3: *F*(3, 392) = 19.86, *p* < 0.001, ${\eta }_{p}^{2}=0.13$, E4: *F*(3, 385) = 16.84, *p* < 0.001, ${\eta }_{p}^{2}=0.12$). We also observe a main effect for perturbation magnitude, with the perceptual bias growing with *ϵ* (E2: *F*(3, 1155) = 10.73, *p* < 0.001, ${\eta }_{p}^{2}=0.03$, E3: *F*(3, 1176) = 22.08, *p* < 0.001, ${\eta }_{p}^{2}=0.05$, E4: *F*(3, 1155) = 17.48, *p* < 0.001, ${\eta }_{p}^{2}=0.04$). A reliable interaction occurs between the target class and perturbation magnitude for E3 and E4, reflecting a larger slope with *ϵ* for some classes than others (E2: *F*(9, 1155) = 1.78, *p* = 0.067, ${\eta }_{p}^{2}=0.01$; E3: *F*(9, 1176) = 2.55, *p* < 0.01, ${\eta }_{p}^{2}=0.02$, E4: *F*(9, 1155) = 4.25, *p* < 0.001, ${\eta }_{p}^{2}=0.03$). See Supplementary Tables  1 and 12 for a summary of statistics from Experiments 1–5. Nonetheless, performing separate ANOVAs for each adversarial target class, every one of the four conditions in each of the three experiments yields a reliable above-chance bias (see Supplementary Note 2).

Across Experiments 2–4, the per-image human perceptual bias is significantly positively correlated with the bias of a black box ANN i.e., a model that was not used in generating perturbations. (E2: Spearman’s *ρ*(1534) = 0.13, *p* < 0.001, E3: *ρ*(1534) = 0.29, *p* < 0.001, E4: *ρ*(1534) = 0.18, *p* < 0.001). Perturbation magnitudes varied from 2 to 16, smaller than has previously been studied with human participants, and similar in magnitude to perturbations used in adversarial machine learning research. Surprisingly, even a perturbation of 2 pixel intensity levels (on a 0-255 scale) is sufficient to reliably bias human perception (With Bonferoni corrected *p* values, E2: *t*(388) = 3.54, *p* = 0.002, Cohen’s *d* = 0.18,two-tailed; E3: *t*(395) = 3.95, *p* < 0.001, Cohen’s *d* = 0.2,two-tailed; E4: *t*(388) = 3.45, *p* = 0.002, Cohen’s *d* = 0.18, two-tailed).

We further tested whether there exists a simpler explanation for observing the effect in our main experiment. First, we investigate whether the effect in experiment 4 is driven by a few outlier stimuli; we analyzed the distribution of per-stimulus perceptual bias (averaged across subject responses to a given stimulus) for all conditions in this experiment by performing the Shapiro-Wilk test for normality and found no credible evidence to reject the hypothesis that the distribution of perceptual bias across stimuli is normal for almost all the conditions (see Supplementary Table  6). Second, we find no credible evidence that participants are more sensitive to perturbations that are highly salient (e.g., textures painted into a uniform background such as the sky) than to ones that are less salient, as measured by the structural similarity index, MS-SSIM^36^ (Supplementary Note 2). Third, participants make relatively few errors in a direct classification task involving single adversarial images, even with *ϵ* = 16, indicating that the perturbations are not altering the ostensible image class (Supplementary Note 3). Fourth, we conducted shuffling analyses, where the shuffling procedure eliminated all the effects, suggesting that the effects that we observed are robust and highly unlikely to occur by chance (Supplementary Note 4).

Each of Experiments 2–4 has a particular strength, but on its own, each has a potential confound. The strength of Experiment 2 is that participants are asked to make an intuitive judgment (e.g., which of two perturbed cat images is more cat-like); however, Experiment 2 allows the possibility that adversarial perturbations cause an image to be more or less cat-like simply by sharpening or blurring the image. The strength of Experiment 3 is that we match all statistics of the perturbations being compared, not just the maximum magnitude of the perturbations. However, matching perturbation statistics does not ensure that the perturbations are equally perceptible when added to the image; consequently, participants might have chosen on the basis of image distortion. In Supplementary Fig. 10, we present a control experiment showing that indeed the A*↑* images are more perceptually distorted, but we further show that judgments in Experiment 3 are not based on perceived distortion. The strength of Experiment 4 is that it proves that participants are sensitive to the question being asked because the same image pair (*A↑* and ${A}^{{\prime} }\uparrow$) yields systematically different responses depending on the question asked (‘more *A*-like’ or ‘more ${A}^{{\prime} }$-like’). However, Experiment 4 requires participants to answer a seemingly nonsensical question (e.g., which of two omelet images looks more cat-like?), leading to potential variability in how the question is interpreted.

Taken together, Experiments 2–4 provide converging evidence that the subordinate adversarial signals that strongly influence ANNs also bias humans in the same direction, even when the perturbation magnitudes are very small and when viewing times are unconstrained. Small perturbations were intriguing to the researchers who first explored the adversarial examples phenomenon on ANNs because of the dramatic impact they had on machine decision and the presumption that they would be imperceptible to humans^25^. Further, extended viewing times—the circumstances of natural perception—are key to the existence of practical consequences of adversarial perturbations.

### What properties of the ANN are critical to perturbation effectiveness?

Having shown human susceptibility to adversarial examples, we turn to investigate the particular ANN properties that influence this susceptibility. We utilize two model classes, convolutional and self-attention architectures. Convolutional networks^5,37^ are the dominant architecture used in computer vision and in modeling the human visual system^38^; they incorporate strong inductive biases such as local receptive fields and approximate translation equivariance. Convolutional models apply static local filters across the visual field and build a hierarchy of representations by repeating this operation multiple times, mimicking the hierarchy in the ventral pathway of the visual cortex^39^. Convolutional networks are inspired by the primate visual system^5,6^ with convolution and pooling operations connecting to the simple and complex cells in the visual system^40^. Recently, a new class of architectures has arisen for computer vision that incorporates mechanisms of self attention^41–44^. Originally, these mechanisms were designed to tackle problems in natural-language processing e.g., transformers^45^; and thus received no explicit architectural inspiration from the visual system. The self-attention operation determines a weighting for embeddings of different tokens or words to obtain the next level of representation in the network hierarchy. To adapt these models to image processing, the image is typically divided into non-overlapping patches and then these patches are processed as if they are a sequence of words in a sentence^43,44^. The main operation in self-attention models is nonlocal, allowing for global communication across the entire image space. Self-attention models achieve state-of-the-art performance and have some intriguing differences from convolutional models, including the fact that self-attention models have a relatively greater bias toward shape features than texture features as compared to convolutional models^46,47^, consistent with human vision. The errors produced by self-attention models better match human error patterns than errors produced by convolutional models^46^, possibly due to their ability to extract shape features.

We constructed two alternative models, one convolutional and one based on self attention, trained on the same data. Both models achieve comparable top-1 and near state-of-the-art classification accuracy on ImageNet (86.3% and 86.6%, respectively). We conducted a version of Experiment 4 using perturbations generated by either convolutional or self-attention models. Human perception is biased in the predicted direction in both conditions (convolutional: *t*(380) = 3.91, *p* < 0.001, Cohen’s *d* = 0.2, 95% CI of difference between means = [0.01, 0.02], two-tailed; self attention: *t*(380) = 5.98, *p* < 0.001, Cohen’s *d* = 0.31, 95% CI of difference between means = [0.02, 0.03], two-tailed), indicating that both models are aligned with human perception. We also observed the presence of stimuli that affected human perception collectively across groups of non-overlapping participant pools with varied degrees of effectiveness (See Supplementary Fig. 4). Because structural differences between convolutional and self-attention models lead to somewhat different image representations^48^, we ask whether one or the other is better aligned. We find little credible evidence for a difference between the bias in the two conditions (0.515 versus 0.525, *t*(379) = 1.87, *p* = 0.062, Cohen’s *d* = 0.13, 95% CI of difference between means = [0.0, 0.02], two-tailed; see Supplementary Fig. 5).

**Fig. 4:**
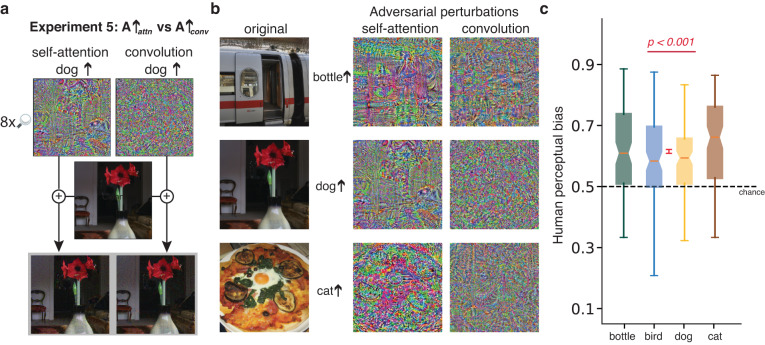
Experiment 5: Participants are more sensitive to adversarial images produced by self-attention ANNs than convolutional ANNs. **a** Adversarial perturbations (*ϵ* = 16) of an image of true class *T* are obtained from either self-attention or convolutional ANNs that increase the model’s confidence in adversarial class *A*, *A* ≠ *T*. Participants are asked to judge which of the two adversarial images are more like an instance of class *A*. **b** Examples of original images and corresponding perturbations produced by the two models toward specific adversarial classes. The perturbations produced by the self-attention model have a more apparent structure. Example images in a and b are obtained from the Microsoft COCO dataset^62^ and OpenImages dataset^63^. **c** Box plots (same convention as Fig. 2c) indicating the probability that *n* = 396 independent participants (cat *n* = 100, dog *n* = 98, bird *n* = 100, bottle *n* = 98) prefer adversarial images produced by self-attention over convolutional ANNs for each of four classes. Participants reliably prefer adversarial images produced by self-attention over convolutional ANNs (*t*(396) = 18.25, *p* < 0.001, Cohen’s *d* = 0.91, 95% CI of difference between means = [0.1, 0.13], two-tailed test). Mean across conditions is shown as a red point with ±1 SE errorbar.

As a stronger assessment of the relative effectiveness of the two representations, we conducted a further experiment requiring participants to select between adversarial images generated by the two models. Using each of the models, we generated adversarial examples from an image of a true class *T* and perturbed toward adversarial class *A*, denoted *A↑*_*conv*_ and *A↑*_*attn*_. We presented matched pairs of adversarial images—*A↑*_*conv*_ and *A↑*_*attn*_—to participants and asked which of the two images is more *A*-class like (Fig. 4a). Participants are more likely to choose adversarial images from the self-attention model than the convolution model being *A*-class like (*t*(396) = 18.25, *p* < 0.001, Cohen’s *d* = 0.91, 95% CI of difference between means = [0.1, 0.13], two-tailed); see Fig. 4c. To rule out a trivial low-level explanation for the preference, we verified that no credible evidence exists of a difference in the luminance and contrast distributions of convolution and self-attention stimuli (Supplementary Fig. 2).

The more interesting hypothesis for the selection preference, suggested by the literature^46,47^, is that image representations in human perception better match representations obtained by self-attention models. We conducted further analyses to understand the nature of the representational differences between self-attention and convolutional models. We quantified the shape bias of the two models we used in this experiment using the Stylized-ImageNet dataset^23^ and found that indeed the self-attention model shows more shape bias (46.9%) than the convolution model (41.7%) (Supplementart Tables 14 and 15). Further, the self-attention model is more robust to image noise corruptions (Supplementary Tables  16 and  17) presumably as a result of its greater reliance on shape features.

Inspecting the perturbations produced by self-attention models, they appear to have more structure in that the perturbations are aligned with edges in the original image (e.g., see Fig. 4b). To formalize a measure of edge strength, we use an automatic procedure to extract edges from the adversarial perturbations and sum the evidence for edges across image space. Perturbations generated by the self-attention model contained significantly more evidence for edges than those generated by the convolutional model (*t*(415) = 9.8, *p* < 0.001, Cohen’s *d* = 0.68, 95% CI of difference between means = [0.01, 0.02], two-tailed). Further, on an individual trial basis, human preference for one image of a pair is correlated with the difference in edge strengths of the perturbations forming the pair (Spearman’s *ρ*(413) = 0.16, *p* = 0.001, 95% CI of *ρ* = [0.06, 0.25]; see Supplementary Fig. 3). This correlation indicates that participants are in fact sensitive to structural changes made to images, even when those structural changes are subtle (see, for example, Fig. 4a).

## Discussion

In this work, we showed that subtle adversarial perturbations, designed to alter the classification decisions of artificial neural networks, also influence human perception. This influence is revealed when experimental participants perform forced-choice classification tasks, whether image exposures are brief or extended (Figs. 2 and 3, respectively). Reliable effects of adversarial perturbations are observed even when the perturbation magnitudes are so tiny that there is little possibility of overtly transforming the image to be a true instance of the adversarial class. Adversarial perturbations have intrigued the academic community—as well as the broader population—because the perturbations appear perceptually indistinguishable from noise, in that one might expect them to degrade human perceptual accuracy but not bias perception systematically in the same direction as neural networks are biased.

Even though our adversarial manipulations induced a reliable change in human perception, this change is not nearly as dramatic as what is found in artificial neural nets, which completely switch their classification decisions in response to adversarial perturbations. For example, in Experiment 4, whereas our participants chose the response consistent with the adversarial perturbation on 52% of trials (for epsilon = 2), a black-box attack on a neural net showed a two-alternative choice preference consistent with the adversarial perturbation on 85.3% of trials. (A black-box attack refers to the fact that the neural net used for testing is different than the model used to generate the adversarial perturbation in the first place.)

One minor factor for the weak human response is that on any trial where participants are inattentive to the stimulus, the choice probability will regress to the chance rate of 50%. The more substantive factors reflect fundamental differences between humans and the type of neural networks that are used to obtain adversarial images. While we cannot claim to enumerate all such differences, four points stand out in the literature: (1) Even with millions of training examples, the data that neural network classifiers are exposed to do not reflect the richness of naturalistic learning environments. Consequently, image statistics learned by neural nets are likely deficient. Bhojanapalli et al.^49^ and Sun et al.^50^ found that as training corpus size increases, neural networks do show improved robustness to adversarial attacks; this robustness is observed for both convolutional and self-attention models and for a variety of attacks. (2) The models used for generating adversarial perturbations are trained only to classify, whereas human vision is used in the service of a variety of goals. Mao et al., 2020^51^ indeed found that when models are trained on multiple tasks simultaneously, they become more robust to adversarial attacks on the individual tasks. (3) Typical neural networks trained to classify images have at best an implicit representation of the three-dimensional structure and of the objects composing a visual scene. Recent vision ANN models have considered explicit figure-ground segmentation, depth representation, and separate encoding of individual objects in a scene. Evidence points to these factors increasing adversarial and other forms of robustness^52–54^. (4) Common ANN architectures for vision are feedforward, whereas a striking feature of the visual cortex is the omnipresence of recurrent connectivity. Several recent investigations have found improvements in adversarial robustness for models with lateral recurrent connectivity^55^ and reciprocal connectivity between layers^56^.

What is the explanation for the alignment found between human and machine perception? Because both convolutional and self-attention ANNs—models with quite different architectural details—are able to predict human choice (Supplementary Fig. 5), the alignment cannot primarily be due to ANNs having coarse structural similarities to the neuroanatomy of the visual cortex. Indeed, if anything, self-attention models—whose global spatial operations are unlike those in the visual system—are better predictors, though this trend was not statistically reliable. Nonetheless, in a direct comparison between adversarial stimuli designed for the two models, experimental participants strongly choose images that fool self-attention models over images that fool convolutional models (Fig. 4). Self-attention models have a greater tendency to be fooled by images that are perturbed along contours or edges, and we found a reliable correlation between the presence of contour or edge perturbations and the preference for that adversarial image. An interesting topic for future research would be to explore other techniques that better align human and machine representations^57,58^ and to utilize human susceptibility to adversarial perturbations as a diagnostic of that alignment. This study did not include data on sex and age, which may be contributing factors to the susceptibility of humans to adversarial perturbations. Future studies may address this limitation and explore the impact of sex and age.

Taken together, our results suggest that the alignment between human and machine perception is due to the fact that both are exquisitely sensitive to subtle, higher-order statistics of natural images. This further supports the importance of higher order image statistics to neural representations^59^. Progress in ANN research has resulted in powerful statistical models that capture correlation structures inherent in an image data set. Our work demonstrates that these models can not only be exploited to reveal previously unnoticed statistical structure in images beyond low-order statistics but that human perception is influenced by this structure.

## Broader impact

In this work, we study the human classification of images that have been adversarially perturbed using ML models. A stark difference between human and machine perception highlighted in this work is that adversarial perturbations affect the identification of image class far more in machines than in humans. This observation encourages research in machine learning to look closely at potential solutions for improving the brittleness of models, along the lines we mention in the previous section. Further, our discovery of shared sensitivities between humans and machines may encourage human vision research to explore different classes of architectures for modeling the visual system (e.g., self-attention architectures), and may drive further efforts to identify shared sensitivities. These activities may provide insight into mechanisms and theories of the human visual system and may lead away from the use of CNNs as the primary functional model of biological vision. Our work also cautions the computer vision and deep learning communities on the value of formal experimental studies in addition to relying on intuition and self reflection.

In terms of potential future implications of this work, it is concerning that human perception can be altered by ANN-generated adversarial perturbations. Although we did not directly test the practical implications of these shared sensitivities between humans and machines, one may imagine that these could be exploited in natural, daily settings to bias perception or modulate choice behavior. Even if the effect on any individual is weak, the effect at a population level can be reliable, as our experiments may suggest. The priming literature has long suggested that various stimuli in the environment can influence subsequent cognition without individuals being able to attribute the cause to the effect^60^. The phenomenon we have discovered is distinguished from most of the past literature in two respects. First, the stimulus itself—the adversarial perturbation—may be interpreted simply as noise. Second, and more importantly, the adversarial attack—which uses ANNs to automatically generate a perturbed image—can be targeted to achieve a specific intended effect. While the effects are small in magnitude, the automaticity potentially allows them to be achieved on a very large scale. The degree of concern here will depend on the ways in which adversarial perturbations can influence impressions. Our research suggests that, for instance, an adversarial attack might make a photo of a politician appear more cat-like or dog-like, but the question remains whether attacks can target non-physical or affective categories to elicit a specific response, e.g., to increase perceived trustworthiness. Our results highlight a potential risk that stimuli may be subtly modified in ways that may modulate human perception and thus human decision making, thereby highlighting the importance of ongoing research focusing on AI safety and security.

## Methods

### Stimuli

Adversarial images used as stimuli in all experiments were generated using an ensemble of ANN models which are listed along with their ImageNet classification performance in Supplementary Tables. 19 and 20. Our stimuli were created to alter the ANN ensemble prediction score for sets of ImageNet classes that we refer to as *coarse classes*. Methods Section “Coarsening of object categories” describes the coarsification procedure and adversarial classes used in our experiments. Images are separately generated for each level (*ϵ*) of perturbation. For Experiment 1, we generated adversarial perturbations with *ϵ* = 32 (out of 256 pixel intensity levels). For experiments 2–5, we generated adversarial stimuli at four perturbation magnitudes, *ϵ* ∈ {2, 4, 8, 16}. For experiment 1, we added adversarial perturbations to images from the ImageNet dataset^61^. For Experiments 2–5, adversarial perturbations were added to a collection of images obtained from the Microsoft COCO dataset^62^ and OpenImages dataset^63^. Our use of the above-mentioned image datasets to create our stimuli was in line with their terms of use. The image resolution used for Experiments 1–4 was 256 × 256 pixels and 384 × 384 pixels for Experiment 5.

### Participants

Experiment 1 included 38 participants with normal or corrected vision. Participants gave informed consent and were awarded reasonable compensation for their time and effort. Participants were recruited from our institute but were not involved in any projects with the research team. Experiment 1 control (i.e., Experiment SI-5) included 50 participants recruited from an online rating platform^64^. For Experiments 2–5, we performed psychophysics experiments using an online rating platform. In each experimental condition, approximately 100 participants were recruited to participate in the task (see Supplementary Table 18 for the exact number). No statistical method was used to predetermine the number of participants, but the sample size was decided to be comparable to that used in previous similar studies^14,33^. Participants received compensation in the range of $8–$15 per hour based on the expected difficulty of the task. No sex or age information was gathered from the participants for all our studies. Our participants were all located in North America and were financially compensated for their participation. We excluded participants if they were not engaged in the task, as assessed using randomly placed catch trials with an unambiguous answer (e.g., pairing an unperturbed dog image with a cat image and asking which image is more cat-like). If a participant failed one catch trial for Experiments 2, 3, and 5, or two catch trials for Experiment 4, the task automatically terminated and their data was not analyzed.

### Experiment structure

For Experiment 1, participants sat on a fixed chair 61 cm away from a high refresh-rate computer screen (ViewSonic XG2530) in a room with dimmed light to classify brief, masked presentations of adversarial images. Participants classified images that appeared on the screen into one of two classes by pressing buttons on a response time box (LOBES v5/6:USTC) using two fingers on their right hand. The assignment of classes to buttons was randomized for each experiment session. Each trial started with a fixation cross displayed in the middle of the screen for 500 − 1000 ms. After the fixation period, an image of fixed size 15.24 cm × 15.24 cm (14. 2° visual angles) was presented briefly at the center of the screen for a period of 63 ms (71 ms for some sessions). The image was followed by a sequence of ten high contrast binary random masks, each displayed for 20 ms (see example in Fig. 2a). Participants pressed one of two buttons to classify the object present in the image. The waiting period to start the next trial was of the same duration whether participants responded quickly or slowly. Each participant’s response time was recorded by the response time box relative to the image presentation time (monitored by a photodiode). In the case where a participant pressed more than one button in a trial, only the class corresponding to their first choice was considered. Each participant completed between 140 and 950 trials.

For Experiments 2–5, Participants performed an extended viewing two-alternative forced-choice task where they saw a pair of images that appeared on the screen and chose the one that looked more like it belonged to a queried target class. In each experiment trial, participants were shown a question above the stimuli which read, “Which image looks more target-like?”, where the target was a cat, bottle, etc. (The complete set of categories is presented in Fig. 3a). Two buttons labeled ‘left’ and ‘right’ were placed below the stimulus pair. Participants used the keyboard keys F and J to select the left and right responses, respectively. (Participants were required to use a computer and not a mobile device to participate.) After a response was made, the images and buttons disappeared followed by an inter-trial-interval (ITI) screen. This ITI screen contained a ‘next trial’ button with the following text: “Press SPACEBAR to move to the next trial.” Participants advanced in the task at their own pace. Individuals were asked to use their own perceptual judgment to make this decision and were informed that their responses will be compared to a machine doing the same task. Each participant completed 104 experiment trials. 8 out of these 104 trials were catch trials with a clear solution placed randomly to measure user engagement. We include a snapshot of the exact experiment description used in our tasks in Supplementary Figs. 6–9.

### ANN models used to create adversarial perturbations

We created adversarial attacks on ANN models trained on a large collection of natural images to classify images into objects. We describe here the specific models used along with training strategies and datasets.

For experiment 1, we used an ensemble of 10 ANN models trained on the ImageNet dataset^61^ to create adversarial perturbations; these models are listed in Supplementary Table 19. For experiments 2–4, we used an ensemble of 6 highly accurate ANNs trained, and calibrated (See Supplementary Table  22 for calibration temperature of these ANNs) on the ImageNet dataset to create adversarial perturbations; these models are listed in Supplementary Table  20. In order to match the initial visual processing of ANN models with human vision, we added an artificial ‘retinal blurring’ layer described in Supplementary Note 1 and Supplementary Algorithm 1 that mimics processing done by primates fovea. However, that choice was not essential and we could not detect a reliably strong increase in human perceptual bias from adding the retinal blurring layer. For experiment 5, we constructed two alternative ANN models (one based on convolution and one based on the self-attention operation) with which adversarial attacks were created. These ANNs were trained on the same data from the in-house JFT-300M^44^ dataset and finetuned on ImageNet. For the former, we used an ensemble of the ResNet-101 and ResNet-200 networks (BiT models described in^44^) and for the latter, we used an ensemble of the ViT-L16 and ViT-B32 networks^44^ respectively. We show in Supplementary Table 21 the accuracy of these models on the ImageNet validation set.

Ensembling predictions from a set of ANN models refers to the process of mathematically combining predictions of an input image from more than one ANN model. This is a common practice used in adversarial machine learning to generate adversarial attacks that transfer across ANN models^65^. We perform a simple aggregation of prediction scores from the ensemble’s individual models by taking an average of the unnormalized predictions (aka logits) across the ANN models. This aggregation is related to the geometric mean of the ANN prediction probability.

### Coarsening of object categories

The ImageNet dataset consists of 1000 fine object categories such as breeds of dogs that a typical human participant may not be familiar with. For this reason, we compute predictions corresponding to groups of the fine object categories that may be more familiar to the experiment participants. We group a subset of the fine classes into one of nine common object categories (sheep, dog, cat, elephant, bird, chair, bottle, truck, and clock) we refer to as coarse categories (coarse categories used in our experiments along with ImageNet support can be found in SupplementaryTable 24). For example, we aggregate predictions from all 120 ImageNet classes that correspond to various dog breeds into a single ‘dog’ coarse category. These coarse categories were chosen arbitrarily by Geirhos et al.^23^ in order to roughly balance natural and human-made categories. Let *S*_*i*_ be the score assigned by our ensemble to a fine ImageNet category *i* (i.e., the value of the *i*^*th*^ unnormalized prediction score; aka logit) and *c* be a coarse category. We compute the unnormalized prediction score to a coarse category *c* as:

1 $${S}_{c}=\left(\log \mathop{\sum}\limits_{i\in c}\exp {S}_{i}\right)-\left(\log \mathop{\sum}\limits_{j\notin c}\exp {S}_{j}\right)$$

This score reflects the logit of the binary classification model that defines the probability of the existence of the coarse category *c*.

### Perturbation generation algorithm

We use the iterative Fast Gradient Sign Method (iFGSM) technique^66^, an iterative adversarial attack method, to create targeted or untargeted adversarial attacks on the ANN ensemble. iFGSM optimizes a small perturbation to the input image by iteratively perturbing the image using information from the image gradient corresponding to minimizing an adversarial objective function.

The adversarial objective function corresponding to a targeted attack towards target class *y* on input *X* is the binary cross entropy loss with label *y*:

2 $${J}_{targeted}({{{{{{{\bf{X}}}}}}}},\,y)=-\log ({P}_{ens}(y|{{{{{{{\bf{X}}}}}}}}))$$

Similarly, the adversarial objective function corresponding to an untargeted adversarial attack reducing the prediction confidence of class *y* for input *X* is the binary cross entropy loss with label ${\bar{y}}$:

3 $${J}_{untargeted}({{{{{{{\bf{X}}}}}}}},\,y)=-\log ({P}_{ens}(\bar{y}|{{{{{{{\bf{X}}}}}}}}))=-log(1-{P}_{ens}(y|{{{{{{{\bf{X}}}}}}}}))$$

The following equation then outlines the iterative procedure used in combination with *J*_*targeted*_ or *J*_*untargeted*_ to create an adversarial attack that increases or decreases prediction confidence on a target class *y* respectively.

4 $${\tilde{{{{{{{{\bf{X}}}}}}}}}}_{i}={\tilde{{{{{{{{\bf{X}}}}}}}}}}_{i-1}+\alpha \times {{{{{{{\rm{sgn}}}}}}}}({{{{{{{{\boldsymbol{\nabla }}}}}}}}}_{{{{{{{{\bf{X}}}}}}}}}(-J({{{{{{{\bf{X}}}}}}}},\,y)))$$

We constrained adversarial perturbations created with iFGSM using the *L*_*∞*_ norm of the perturbation (∣∣**X**_**adv**_ − **X**∣∣_*∞*_≤*ϵ*); we restrict the adversarial perturbations by clipping all perturbed image pixels with intensity less than **X** − *ϵ*, and greater than **X** + *ϵ* as follows:

5 $${{{{{{{{\bf{X}}}}}}}}}_{i}\,=\,{{{{{{{\rm{clip}}}}}}}}({\tilde{{{{{{{{\bf{X}}}}}}}}}}_{i},{{{{{{{\bf{X}}}}}}}}-\epsilon,{{{{{{{\bf{X}}}}}}}}+\epsilon )$$

The above procedure is performed iteratively from *i* ∈ {1, …, *n*} until the final adversarial image **X**_*n*_ is computed.

Viewing conditions for human raters could vary significantly (e.g., raters could be viewing images from different angles, on monitors that have different sizes, or while sitting at various distances away from the monitor), which may interfere with the experiment. To address this problem, we created adversarial images that are largely invariant to the change in these viewing conditions. To achieve this invariance, we modeled the change in viewing condition as the geometric transformation of the image (e.g., different rotation, scale, and translation). We compute adversarial examples that are robust to image transformations by sampling random geometric transformations applied to the original image at each step of the perturbation algorithm (rotation *θ* ~ U(0, *π*/6), scale *s*_*x*_, *s*_*y*_ ~ U(0.5*L, *L*), and translation *t*_*x*_, *t*_*y*_ ~ U(-*L*/4, *L*/4)) where *L* represents the image width in pixels (same as height as we use square images) of our stimuli. Let *t* ∈ *T* be an identity-preserving geometric image transformation that is differentiable and **X** be the input image to be perturbed to be classified as *y*_*target*_. We have the following optimization problem to solve to compute an adversarial image with invariance to geometric transformations.

6 $$\mathop{{{{{{\rm{arg}}}}}}\,{{{{{\rm{max}}}}}}}\limits_{X^{\prime}} {\mathop{\mathbb{E}}\limits_{t \in T}} [\log(P(y_{target}|t({{{{{\mathbf{X}}}}}}^{\prime})))]\quad {{{{{{\rm{s}}}}}}.{{{{{\rm{t}}}}}}.}\,||{{{{{\mathbf{X}}}}}}^{\prime} - {{{{{\mathbf{X}}}}}}||_\infty \leq \epsilon$$

### Class subsampling for experiment 4 and 5 stimuli

Experiments 4 and 5 are different in nature than the rest of the experiments in that we explicitly ask humans to choose an image from a pair of adversarial perturbations towards two different target classes. In this case, there exists a significant difference in ImageNet support corresponding to each individual coarse class in the pair, e.g. there are 120 ‘dog’ classes vs 7 ‘bottle’ classes in ImageNet. In order to prevent such differences from causing a hidden bias in human response, we randomly sampled a subset of the classes to compute coarse target class score for the class that had larger ImageNet support. Let *n*_*A*_ and ${n}_{{A}^{{\prime} }}$ be the number of ImageNet classes corresponding to target classes *A* and ${A}^{{\prime} }$ that are paired in an experiment. For each image, we randomly subsample $\min ({n}_{A},{n}_{{A}^{{\prime} }})$ fine ImageNet classes corresponding to target class *A* (and third class ${A}^{{\prime} }$); these fine classes are then used to for computing the coarse score for target class *A* (or class ${A}^{{\prime} }$) using Eq. (1), which is used in the adversarial objective function that is optimized by the algorithm.

### Edge feature analysis

In order to test whether adversarial perturbations from self-attention ANN ensemble contain more shape information compared to those from the convolutional ANN ensemble, we conducted an edge feature analysis by comparing the number of active edge features in image pairs that appeared in Experiment 5. We apply Bilateral filtering – a non-linear filtering technique known to blur an image and reduce noise while respecting strong edges^67^ – to the adversarial perturbations corresponding to adversarial images generated by the two models. We detected strong edges by applying a Canny edge detector algorithm to the output of Bilateral filtering, resulting in a binary map of unit-length edge elements in this perturbation. We use the absolute count of the number of these edge elements as the “edge strength” corresponding to this adversarial perturbation.

### Reporting summary

Further information on research design is available in the Nature Portfolio Reporting Summary linked to this article.

### Supplementary information

Supplementary Information

Reporting Summary

## Data Availability

For our experiments’ stimuli, we reused images from ImageNet^61^, Microsoft COCO^62^, and OpenImages^63^. Images displayed in this manuscript are covered by Creative Commons BY 2.0 Attributions license (CC-BY2.0). We list the source images in Supplementary Note 5. The human participant responses generated and/or analysed during the current study are available at https://osf.io/dnmkw/^68^. Experiment 1 was originally presented in Elsayed et al., 2018^69^.

## Code availability

Code packages for generating experiments in HTML, Javascript, and jQuery are available at https://osf.io/dnmkw/^68^. We adapted helper packages made publicly available by Zhou & Firestone, 2019^33^. Python notebooks for generating results are also included in https://osf.io/dnmkw/.

## References

[1] Krizhevsky A, Sutskever I, Hinton GE (2012). Imagenet classification with deep convolutional neural networks. Adv. Neural Inf. Proces. Syst..

[2] Collobert R (2011). Natural language processing (almost) from scratch. J. Mach. Learn. Res..

[3] Lee, J., Hwangbo, J., Wellhausen, L., Koltun, V. & Hutter, M. Learning quadrupedal locomotion over challenging terrain. *Sci. Robot.* **5**, eabc5986 (2020). 10.1126/scirobotics.abc5986 33087482

[4] von Neumann, J. *The Computer and the Brain*. The Silliman Memorial Lectures Series (Yale University Press, 1958). https://yalebooks.yale.edu/book/9780300181111/computer-and-brain.

[5] Fukushima K (1980). Neocognitron: a self-organizing neural network model for a mechanism of pattern recognition unaffected by shift in position. Biol. Cybern..

[6] Fukushima K, Miyake S (1982). Neocognitron: a new algorithm for pattern recognition tolerant of deformations and shifts in position. Pattern Recog..

[7] Khaligh-Razavi S-M, Kriegeskorte N (2014). Deep supervised, but not unsupervised, models may explain it cortical representation. PLoS Comput. Biol..

[8] Yamins DL (2014). Performance-optimized hierarchical models predict neural responses in higher visual cortex. Proc. Natl. Acad. Sci..

[9] Cadieu CF (2014). Deep neural networks rival the representation of primate it cortex for core visual object recognition. PLoS Comput. Biol..

[10] Kriegeskorte N (2015). Deep neural networks: a new framework for modeling biological vision and brain information processing. Ann. Rev. Vision Sci..

[11] Yamins DLK, DiCarlo JJ (2016). Using goal-driven deep learning models to understand sensory cortex. Nat. Neurosci..

[12] Serre T (2019). Deep learning: the good, the bad, and the ugly. Ann. Rev. Vision Sci..

[13] Berardino, A., Ballé, J., Laparra, V. & Simoncelli, E. Eigen-Distortions of Hierarchical Representations. In *Proceedings of the 31st International Conference on Neural Information Processing Systems*. **30**, 3533–3542 (Curran Associates Inc., 2017).

[14] Kar, K., Kubilius, J., Schmidt, K., Issa, E. B. & DiCarlo, J. J. Evidence that recurrent circuits are critical to the ventral stream’s execution of core object recognition behavior. *Nat. Neurosci.* **22**, 974–983 (2019). 10.1038/s41593-019-0392-5 PMC8785116 31036945

[15] Kim B, Reif E, Wattenberg M, Bengio S, Mozer MC (2021). Neural networks trained on natural scenes exhibit gestalt closure. Comput. Brain Behav..

[16] Kubilius, J., Bracci, S. & Op de Beeck, H. P. Deep neural networks as a computational model for human shape sensitivity. PLoS Comput. Biol. 12, 1–26 (2016). 10.1371/journal.pcbi.1004896 PMC4849740 27124699

[17] Mozer, M. C.*The perception of multiple objects: a connectionist approach* (MIT Press, Cambridge, MA, 1991).

[18] Rumelhart, D. E., McClelland, J. L. & Group, P. R. (eds.) *Parallel Distributed Processing: Explorations in the Microstructure of Cognition, Vol. 2: Psycholog. Biol. Models* (MIT Press, Cambridge, MA, USA, 1986).

[19] Wenliang LK, Seitz AR (2018). Deep neural networks for modeling visual perceptual learning. J. Neurosci..

[20] Jacobs RA, Bates CJ (2019). Comparing the visual representations and performance of humans and deep neural networks. Curr. Dir. Psycholog. Sci..

[21] Firestone C (2020). Performance vs. competence in human–machine comparisons. Proc. Natl. Acad. Sci..

[22] Geirhos, R. et al. Generalisation in humans and deep neural networks. In *Thirty-second Annual Conference on Neural Information Processing Systems 2018 (NeurIPS 2018)*, 7549–7561 (Curran, 2019).

[23] Geirhos, R. et al. ImageNet-trained CNNs are biased towards texture; increasing shape bias improves accuracy and robustness. In *International Conference on Learning Representations* (2019).

[24] Geirhos, R. et al. Partial success in closing the gap between human and machine vision. *Adv. Neural Inf. Process. Syst.* **34**, 23885–23899 (2021).

[25] Szegedy, C. et al. Intriguing properties of neural networks. In *2nd International Conference on Learning Representations, ICLR 2014*, Conference Track Proceedings (eds. Bengio, Y. & LeCun, Y.) (Banff, AB, Canada, 2014).

[26] Biggio, B. et al. Evasion attacks against machine learning at test time. In *Machine Learning and Knowledge Discovery in Databases - European Conference, ECML PKDD 2013, Prague, Czech Republic, September 23–27, 2013, Proceedings, Part III*, 387–402 (2013).

[27] Goodfellow, I. J., Shlens, J. & Szegedy, C. Explaining and harnessing adversarial examples. In Bengio, Y. & LeCun, Y. (eds.) *3rd International Conference on Learning Representations, ICLR 2015, San Diego, CA, USA, May 7–9, 2015, Conference Track Proceedings* (2015). http://arxiv.org/abs/1412.6572.

[28] Papernot, N., McDaniel, P. D. & Goodfellow, I. J. Transferability in Machine Learning: from Phenomena to Black-Box Attacks using Adversarial Samples. CoRR abs/1605.07277, (2016).

[29] Ilyas, A. et al. Adversarial Examples Are Not Bugs, They Are Features. In *Advances in Neural Information Processing Systems* (eds. Wallach, H. et al.) vol. 32, 125–136 (Curran Associates, Inc., 2019).

[30] Kim, E., Rego, J., Watkins, Y. & Kenyon, G. T. Modeling biological immunity to adversarial examples. In *Proceedings of the IEEE/CVF Conference on Computer Vision and Pattern Recognition*, 4666–4675 (2020).

[31] Ford, N., Gilmer, J., Carlini, N. & Cubuk, E. D. Adversarial examples are a natural consequence of test error in noise. In *International Conference on Machine Learning*, 2280–2289 (PMLR, 2019).

[32] Han, C., Yoon, W., Kwon, G., Kim, D. & Nam, S. Representation of white-and black-box adversarial examples in deep neural networks and humans: a functional magnetic resonance imaging study. In *2019 International Joint Conference on Neural Networks (IJCNN)*, 1–8 (IEEE, 2019).

[33] Zhou Z, Firestone C (2019). Humans can decipher adversarial images. Nat. Commun..

[34] Dujmović M, Malhotra G, Bowers JS (2020). What do adversarial images tell us about human vision?. Elife.

[35] Yuan, L. et al. Adversarial images for the primate brain. *arXiv preprint arXiv:2011.05623* (2020).

[36] Wang Z, Bovik AC, Sheikh HR, Simoncelli EP (2004). Image quality assessment: from error visibility to structural similarity. IEEE Trans. Image Proces..

[37] LeCun, Y. et al. Generalization and network design strategies. Connectionism in perspective. Zurich, Switzerland, Elsiever 19, 18 (1989).

[38] Lindsay, G. W. Convolutional neural networks as a model of the visual system: Past, present, and future. *J. Cogn. Neurosci.* **33**, 2017–2031 (2021). 10.1162/jocn_a_01544 32027584

[39] Felleman DJ, Van Essen DC (1991). Distributed hierarchical processing in the primate cerebral cortex. Cereb. Cortex (New York, NY: 1991).

[40] Hubel DH, Wiesel TN (1962). Receptive fields, binocular interaction and functional architecture in the cat’s visual cortex. J. Physiol..

[41] Ramachandran, P. et al. Stand-alone self-attention in vision models. *Adv. Neural Inf. Process. Syst.* **32**, 68–80 (2019).

[42] Parmar, N. et al. Image transformer. In *International Conference on Machine Learning*, 4055–4064 (PMLR, 2018).

[43] Chen, M. et al. Generative pretraining from pixels. In *International Conference on Machine Learning*, 1691–1703 (PMLR, 2020).

[44] Dosovitskiy, A. et al. An Image is Worth 16x16 Words: Transformers for Image Recognition at Scale. In *International Conference on Learning Representations* (2021).

[45] Vaswani, A. et al. Attention is all you need. In *Advances in neural information processing systems*, 5998–6008 (2017).

[46] Tuli, S., Dasgupta, I., Grant, E. & Griffiths, T. L. Are Convolutional Neural Networks or Transformers more like human vision? CoRR abs/2105.07197 (2021).

[47] Naseer, M. et al. Intriguing properties of vision transformers. *Adv. Neural Inf. Process. Syst.* **34**, 23296–23308 (2021).

[48] Raghu, M., Unterthiner, T., Kornblith, S., Zhang, C. & Dosovitskiy, A. Do vision transformers see like convolutional neural networks? *Adv. Neural Inf. Process. Syst.* **34**, 12116–12128 (2021).

[49] Bhojanapalli, S. et al. Understanding robustness of transformers for image classification. In *Proceedings of the IEEE/CVF International Conference on Computer Vision 10231–10241* (IEEE Computer Society, 2021).

[50] Sun, K., Zhu, Z. & Lin, Z. Towards understanding adversarial examples systematically: exploring data size, task and model factors. *arXiv preprint arXiv:1902.11019* (2019).

[51] Mao, C. et al. Multitask learning strengthens adversarial robustness. In *Computer Vision–ECCV 2020: 16th European Conference, Glasgow, UK, August 23–28, 2020, Proceedings, Part II 16*, 158–174 (Springer, 2020).

[52] Xiang, C., Qi, C. R. & Li, B. Generating 3d adversarial point clouds. In *Proceedings of the IEEE/CVF Conference on Computer Vision and Pattern Recognition*, 9136–9144 (2019).

[53] Akumalla, A., Haney, S. & Bazhenov, M. Contextual Fusion For Adversarial Robustness. CoRR abs/2011.09526 (2020).

[54] Dittadi, A. et al. Generalization and Robustness Implications in Object-Centric Learning. In *Proceedings of the 39th International Conference on Machine Learning (ICML)* (eds. Chaudhuri et al.) vol. 162, 5221–5285 (PMLR, 2022).

[55] Paiton DM (2020). Selectivity and robustness of sparse coding networks. J. Vis..

[56] Huang, Y. et al. Neural networks with recurrent generative feedback. *Adv. Neural Inf. Process. Syst.* **33**, 535–545 (2020).

[57] Roads, B. D. & Love, B. C. Enriching imagenet with human similarity judgments and psychological embeddings. In *Proceedings of the IEEE/CVF Conference on Computer Vision and Pattern Recognition*, 3547–3557 (2021).

[58] Attarian, I. M., Roads, B. D. & Mozer, M. C. Transforming neural network visual representations to predict human judgments of similarity. In *NeurIPS Workshop on Shared Visual Representations Between Humans and Machines* (2020).

[59] Simoncelli EP, Olshausen BA (2001). Natural image statistics and neural representation. Ann. Rev. Neurosci..

[60] Bargh JA (2016). Awareness of the prime versus awareness of its influence: Implications for the real-world scope of unconscious higher mental processes. Curr. Opinion Psychol..

[61] Deng, J. et al. Imagenet: A large-scale hierarchical image database. In *2009 IEEE conference on computer vision and pattern recognition*, 248–255 (Ieee, 2009). IEEE. 10.1109/CVPR.2009.5206848.

[62] Lin, T.-Y. et al. Microsoft coco: Common objects in context. In *European conference on computer vision*, 740–755 (Springer, 2014). Springer, Cham. 10.1007/978-3-319-10602-1_48.

[63] Kuznetsova, A. et al. The open images dataset v4: Unified image classification, object detection, and visual relationship detection at scale. *IJCV* (2020). Springer Nat. 10.1007/s11263-020-01316-z.

[64] Mason W, Suri S (2012). Conducting behavioral research on amazon’s mechanical turk. Behav. Res. Methods.

[65] Liu, Y., Chen, X., Liu, C. & Song, D. Delving into Transferable Adversarial Examples and Black-box Attacks. In *International Conference on Learning Representations* (2017).

[66] Kurakin, A., Goodfellow, I. & Bengio, S. Adversarial examples in the physical world. In *ICLR’2017 Workshop* (2016). https://arxiv.org/abs/1607.02533.

[67] Tomasi, C. & Manduchi, R. Bilateral filtering for gray and color images. In *Sixth international conference on computer vision (IEEE Cat. No. 98CH36271)*, 839–846 (IEEE, 1998).

[68] Veerabadran, V. et al. Subtle adversarial image manipulations influence both human and machine perception (2022). osf.io/dnmkw. OSF. 10.17605/osf.io/dnmkw. 10.1038/s41467-023-40499-0 PMC10427626 37582834

[69] Elsayed, G. et al. Adversarial Examples that Fool both Computer Vision and Time-Limited Humans. In *Advances in Neural Information Processing Systems* (eds. Bengio, S. et al.) vol. 31, 3914–3924 (Curran Associates, Inc., 2018).

[70] Papernot, N. et al. The limitations of deep learning in adversarial settings. In *2016 IEEE European symposium on security and privacy (EuroS&P)*, 372–387 (IEEE, 2016).

[71] Nguyen, A., Yosinski, J. & Clune, J. Deep neural networks are easily fooled: High confidence predictions for unrecognizable images. In *Proceedings of the IEEE conference on computer vision and pattern recognition*, 427–436 (2015).

[72] Athalye, A., Engstrom, L., Ilyas, A. & Kwok, K. Synthesizing robust adversarial examples. In *International conference on machine learning*, 284–293 (PMLR, 2018).

