# Author Correction: Subtle adversarial image manipulations influence both human and machine perception

**DOI:** 10.1038/s41467-024-44826-x

**Published:** 2024-01-16

**Authors:** Vijay Veerabadran, Josh Goldman, Shreya Shankar, Brian Cheung, Nicolas Papernot, Alexey Kurakin, Ian Goodfellow, Jonathon Shlens, Jascha Sohl-Dickstein, Michael C. Mozer, Gamaleldin F. Elsayed

**Affiliations:** 1 Google, Mountain View, CA USA; 2 Google DeepMind, Mountain View, CA USA; 3 Present Address: Department of Cognitive Science, University of California, San Diego, CA USA; 4 Present Address: University of California, Berkeley, CA USA; 5 Present Address: MIT Brain and Cognitive Sciences, Cambridge, MA USA

**Keywords:** Human behaviour, Visual system, Computer science

Correction to: *Nature Communications* 10.1038/s41467-023-40499-0, published online 15 August 2023

The original version of this Article included incorrect acknowledgements to the sources of Fig. 1c. The following sentence in the original caption to Fig. 1 “Illustration images in this Figure were obtained with permission from^33,35^.” has been changed to “Illustration images in panel (c) were obtained with permission from Papernot et al.^70^, Nguyen et al.^71^, and Athalye et al.^72^, left to right, respectively.”

The following missing references have also been added to the reference list:

[70] Papernot, N. et al. The limitations of deep learning in adversarial settings. In *2016 IEEE European symposium on security and privacy (EuroS&P)*, 372–387 (IEEE, 2016).

[71] Nguyen, A., Yosinski, J. & Clune, J. Deep neural networks are easily fooled: High confidence predictions for unrecognizable images. In *Proceedings of the IEEE conference on computer vision and pattern recognition*, 427–436 (2015).

[72] Athalye, A., Engstrom, L., Ilyas, A. & Kwok, K. Synthesizing robust adversarial examples. In *International conference on machine learning*, 284–293 (PMLR, 2018).

This has been corrected in the PDF and HTML versions of the Article.

