# Supplementary material for: Subtle adversarial image manipulations influence both human and machine perception
Source: Nat Commun. 2023 Aug 15;14:4933. doi: 10.1038/s41467-023-40499-0 (PMC10427626; doi:10.1038/s41467-023-40499-0)
Supplement: Supplementary file 1 — Supplementary Information [file 41467_2023_40499_MOESM1_ESM.pdf]

Supplementary Information

A Supplementary Note 1

Retinal blurring layer:

Let d_{viewer} be the distance (in meters) of the viewer from the display and d_{hw} be the height and width of a square image (in meters). For every spatial position (in meters) $c = (x, y) \in R^2$ in the image we compute the retinal eccentricity (in radians) as follows:

$$\theta(c) = \tan^{-1}\left(\frac{||c||_2}{d_{viewer}}\right) \tag{7}$$

and turn this into a target resolution in units of radians

$$r_{rad}(c) = \min\left(\alpha\theta(c), \beta\right). \tag{8}$$

We then turn this target resolution into a target spatial resolution in the plane of the screen,

$$r_m(c) = r_{rad}(c)\left(1 + \tan^2(\theta(c))\right), \tag{9}$$

$$r_{pixel}(c) = r_m(c) \cdot [\text{pixels per meter}]. \tag{10}$$

This spatial resolution for two point discrimination is then converted into a corresponding low-pass cutoff frequency, in units of cycles per pixel,

$$f(c) = \frac{\pi}{r_{pixel}}, \tag{11}$$

where the numerator is π rather than 2π since the two point discrimination distance r_{pixel} is half the wavelength.

Finally, this target low-pass spatial frequency $f(c)$ for each pixel is used to linearly interpolate each pixel value from the corresponding pixel in a set of low pass filtered images, as described in the following Algorithm 1 (all operations on matrices are assumed to be performed elementwise), We additionally cropped $\mathbf{X}_{retinal}$ to 90% width before use, to remove artifacts from the image edge.

Note that because the per-pixel blurring is performed using linear interpolation into images that were low-pass filtered in Fourier space, this transformation is both fast to compute and fully differentiable.

Algorithm 1 Applying retinal blur to an image

$\mathbf{X}_{img} \leftarrow$ input image
$\mathbf{F}$: image containing corresponding target lowpass frequency for each pixel.
$\mathbf{F} \leftarrow f(c)$
$\tilde{\mathbf{X}} \leftarrow \text{FFT}(\mathbf{X}_{img})$
$\mathbf{G} \leftarrow$ norm of spatial frequency at each position in Y
CUTOFF_FREQS $\leftarrow$ list of frequencies to use as cutoffs for low-pass filtering
for f' in CUTOFF_FREQS **do**
  $\tilde{\mathbf{Y}}_{f'} \leftarrow \tilde{\mathbf{X}} \odot \exp\left(-\frac{G^2}{f^2}\right)$
  $\mathbf{Y}_f \leftarrow \text{InverseFFT}(\tilde{\mathbf{Y}}_{f'})$
end for
$\mathbf{w}(\mathbf{c}) \leftarrow$ linear interpolation coefficients for $F(c)$ into CUTOFF_FREQS $\quad \forall c$
$\mathbf{X}_{retinal}(c) \leftarrow \sum_{f'} w_{f'}(c)\mathbf{Y}_{f'}(c) \quad \forall c$

B Supplementary Note 2

B.1 Per class analysis

In addition to the overall ANOVA with ϵ and target class as independent variables, we conducted ANOVAs for each target class separately. Experiments 2 and 3 each had four target classes (dog, cat, bottle, bird), tested in a between-participant design. Experiment 4 had four target-class pairs (cat-truck, sheep-chair, dog-bottle, elephant-clock), also tested in a between-participant design. For each analysis by class (or class pair), there were approximately 100 participants (see Table Supp.18 for exact counts). For every target class, we find a preference for the adversarial image that is reliably above chance (Table Supp.2). Thus, over the set of classes that we chose in advance, the neural network predicted human preferences for every one. Additionally, for about half the target classes, we find a reliable effect of ϵ (Table Supp.3). We further bolstered this analysis by conducting a more targeted test that focuses on the correlation between perturbation magnitude and human perceptual bias. We computed the Spearman correlation between ϵ and perceptual bias for each participant in each condition and tested whether the correlation was reliably positive. We find that the correlation was significantly positive for 8 of the 12 conditions (Table Supp.4).

B.2 MS-SSIM and perceptual bias analysis

In this section, we examine the relationship between the multi-scale structural similarity (MS-SSIM) [36] and the perceptual bias induced by an adversarial image (i.e., the probability above chance that participants chose the adversarial image). MS-SSIM measures the perceptual similarity of an original image and perturbed image. Thus, MS-SSIM is a measure of saliency of the perturbations and is also a good proxy for homogeneity of image. Since MS-SSIM is a measure that compares original and perturbed images it also necessarily is sensitive to the magnitude of the adversarial perturbations. For the same set of original images, we compute the correlation between the perceptual bias and the MS-SSIM metric computed for adversarial images with perturbations of the same magnitude ϵ. We compute the correlation individually for each value of perturbation magnitude ϵ to focus on the effect of the homogeneity of the original image on perceptual bias rather than perturbation magnitude. At all perturbation magnitudes, we find no evidence of difference in participants' sensitivity to perturbations that are highly salient (e.g., textures painted into a uniform background such as the sky) than to ones that are less salient (Figure Supp.11), as measured by MS-SSIM (Spearman's rank correlation at $\epsilon = 2 : \rho = -0.06$, p = 0.10, 95% CI of ρ=[-0.13, 0.01]; $\epsilon = 4 : \rho = -0.06$, p = 0.10, 95% CI of ρ=[-0.13, 0.01]; $\epsilon = 8 : \rho = -0.05$, p = 0.18, 95% CI of ρ=[-0.12, 0.02]; $\epsilon = 16 : \rho = -0.05$, p = 0.15, 95% CI of ρ=[-0.12, 0.02]). To obtain converging evidence for our conclusion, for each level of ϵ, we further split the images by MS-SSIM into three equal-sized groups (low, medium, and high). We conducted

a one-way ANOVA with image as the random factor and mean bias across participants as the dependent variable. The ANOVA asks whether (1) the overall mean is nonzero, and (2) whether there is a main effect of MS-SSIM group. For each level of ϵ, we failed to detect a significant effect ($\epsilon = 2 : F(2, 765) = 0.988$, $p > 0.35$, $\eta_p^2 = 0.003$; $\epsilon = 4 : F(2, 765) = 0.733$, $p = 0.45$, $\eta_p^2 = 0.002$; $\epsilon = 8 : F(2, 765) = 1.211$, $p = 0.30$, $\eta_p^2 = 0.003$; $\epsilon = 16 : F(2, 765) = 1.271$, $p = 0.28$, $\eta_p^2 = 0.003$). Further, the mean bias is reliably larger than 0 for all perturbation magnitudes ($\epsilon = 2 : F(1, 765) = 5.97$, $p = 0.015$, $\eta_p^2 = 0.01$, 95% CI of mean bias=[0.0, 0.03]; $\epsilon = 4 : F(1, 765) = 18.2$, $p < 0.001$, $\eta_p^2 = 0.02$, 95% CI of mean bias=[0.01, 0.04]; $\epsilon = 8 : F(1, 765) = 80.2$, $p < 0.001$, $\eta_p^2 = 0.09$, 95% CI of mean bias=[0.04, 0.07]; $\epsilon = 16 : F(1, 765) = 107.6$, $p < 0.001$, $\eta_p^2 = 0.12$, 95% CI of mean bias=[0.05, 0.08]).

C Supplementary Note 3

Experiment SI-4: 10-way classification We conducted an additional experiment in which we asked participants to classify adversarially perturbed images. Participants chose a response class from 10 alternatives, one of which was always the true image class (T), one was the adversarial class (A), and one was an alternative class (A'). This experiment included pairs of perturbed images that came from the same source image, one warped from target class T to adversarial class A ($T \to A$) and one to a different class A' ($T \to A'$), all with $\epsilon = 16$. If an adversarial perturbation is affecting the ostensible image class, then one would expect many A responses for a $T \to A$ image but few A responses for a $T \to A'$ image and vice versa. The difference in $T \to A$ and $T \to A'$ error rates is a measure of misclassification corrected for intrinsic ambiguity in the imxage. We identified $\{T \to A, T \to A'\}$ pairs, which individually had been shown to at least three participants. (Fewer than three participants would not provide a meaningful estimate of error rate.) With 310 such pairs, we see a higher response rate for A with $T \to A$ vs. $T \to A'$ (1.6% vs. 0.9%) but this difference is not statistically reliable ($t(309) = 1.83, p = 0.069$, Cohen's d=0.11, 95% Confidence Interval of difference between means=[0.0, 0.2]). It is likely that a better controlled study would have found a difference though, so we further investigated the nature of the manipulated images that yielded high error rates. Figure Supp.12 shows the 10 $T \to A$ images with the highest corrected misclassification rate along with the corresponding original image. Note that (1) only a few images are responsible for the difference in error rates, (2) some images are intrinsically ambiguous or have features in the original image suggestive of class A (e.g., the evidence for a person sitting in the $A =$ chair images), and (3) there is little evidence that the adversarial perturbations "paint in" a canonical instance of of the adversarial class. Because of (2) and (3), none of the images "look like" adversarial class A and so we suspect that there would be few if any A responses if participants were not making a forced-choice selection. To address the possibility whether such a few images were responsible of the main effects we find, we identified 25 in the dataset for which even weak evidence existed of potential misclassification (i.e., given a pair of images in which original class T was perturbed to classes A and A', participants made even one more misclassification of A on $T \to A$ than on $T \to A'$). We then repeated our ANOVA for main Exp. 4 but first excluding those 25 images. The results of this ANOVA were unchanged from those reported in the main paper for Exp. 4: we obtain a main effect of class ($F(3, 385) = 15.7, p < 0.001$, $\eta_p^2 = 0.11$) and a main effect of epsilon ($F(3, 1155) = 19.1, p < 0.001$, $\eta_p^2 = 0.05$). Numerically, the condition means look quite similar suggesting that these misperceived images are unlikely to have driven the effect in Experiment 4. We report 95% Confidence Interval of perceptual bias for the above ANOVA analysis in Table Supp.10.

D Supplementary Note 4

In order to validate the assumption of equal variance across comparison groups in Experiments 1-5, we calculated the variances of the data and found that they were roughly uniform (Table Supp.11). Due to a floor effect inherent in our experiment design, where a systematic bias below 0.5 can not occur (since random choice is at 0.5), we expected and observed a non-normal yet unimodal distribution of the dependent variable's residuals in some of the Experiments (Figure Supp.13 and Table Supp.13, where we used Shapiro-Wilk test to assess the normality of the residuals). However, the mixed-ANOVA analyses we performed are known to be robust to the dependent variable violating the normality assumption. To further confirm that our results are not due to chance, we conducted a nonparametric shuffling analysis on the data from Experiment 1-4. For each experiment, randomly reassigned the factor-level labels and reanalyzed the data. The shuffling procedure eliminated all the effects we observed in our unshuffled data (Table Supp.7). The F score we obtained for the unshuffled data was an extreme outlier in each case, suggesting that the effects of perturbation magnitude and object category that we observed are robust and highly unlikely to occur by chance (Table Supp.8).

E Supplementary Note 5

COCO images displayed in this manuscript are covered by Creative Commons BY 2.0 Attributions license (CC-BY2.0). Below we list all sources for these images.

- Figure 1a, 3b, Supp.10.a: Bear, taken by "Michelle Callahan" on June 12, 2013 available at www.flickr.com/photo.gne?id=9034892699
- Figure 2: Smart Dogs in Vietnam!, taken by "Ian @ ThePaperboy.com" on September 2, 2008 available at www.flickr.com/photo.gne?id=2864552036
- Figure 3a, 3b: FiKittyride01, taken by "Adam Smith" on December 28, 2004 available at www.flickr.com/photo.gne?id=3620403
- Figure 3c: mine, taken by "Missi" on July 8, 2012 available at www.flickr.com/photo.gne?id=8250132140
- Figure 3c: Untitled image taken by "steve p2008" on February 11, 2011 available at www.flickr.com/photo.gne?id=5436959324
- Figure 3c: Railroad Day at Natural Tunnel 2013, taken by "Virginia State Parks" on July 12, 2013 available at www.flickr.com/photo.gne?id=9310473102
- Figure 3c: 17659 - V159DFT, taken by "Mikey" on November 3, 2012 available at www.flickr.com/photo.gne?id=8151925296
- Figure 3c: don't know what possessed me to add a side, taken by "jeffreyw" on March 1, 2010 available at www.flickr.com/photo.gne?id=4399990688

- Figure 4a, 4b: Full bloom, taken by "Robbie Sproule" on January 17, 2010 available at www.flickr.com/photo.gne?id=4283252006
- Figure 4b: Friday Night Pizza, taken by "SteveR-" on October 3, 2008 available at www.flickr.com/photo.gne?id=2914784404
- Figure 4b, Figure Supp.11.b: Arrival in Leipzig, taken by "vxla" on November 26, 2009 available at www.flickr.com/photo.gne?id=4140948175
- Figure Supp.1, Supp.11.a: Zebras, taken by "NeilsPhotography" on August 13, 2003 available at www.flickr.com/photo.gne?id=2349807454
- Figure Supp.11.a: ERJ 145, taken by "Piper" on January 28, 2012 available at www.flickr.com/photo.gne?id=6779312189
- Figure Supp.11.b: yellow_carrots, taken by "istolethetv" on September 10, 2005 available at www.flickr.com/photo.gne?id=121680640
- Figure Supp.12: elephants crossing the chobe river, taken by "abi.bhattachan" on August 23, 2010 available at www.flickr.com/photo.gne?id=4954782165

F Supplementary Tables

Table Supp.1: **Statistics table for all experiments**

Analysis identifier	Test type	Exact n	Degrees of freedom	Test statistic	p value	effect size	95% CI
E1: $A\uparrow$ vs control	Two-tailed t test	91	90	t=4.46	$p < 0.001$	Cohen's d=0.66	Table Supp.12
E2: $T\uparrow$ vs $T\downarrow$– ϵ	2-way ANOVA	1556	(3, 1155)	F=10.73	$p < 0.001$	η_p^2=0.03	Table Supp.25
E2: $T\uparrow$ vs $T\downarrow$– target class	2-way ANOVA	1556	(3, 385)	F=12.94	$p < 0.001$	η_p^2=0.09	Table Supp.2
E2: $T\uparrow$ vs $T\downarrow$– target class x ϵ	2-way ANOVA	1556	(9,1155)	F=1.78	$p = 0.067$	η_p^2=0.01	Table Supp.12
E3: $A\uparrow$ vs control – ϵ	2-way ANOVA	1584	(3, 1176)	F=22.08	$p < 0.001$	η_p^2=0.05	Table Supp.25
E3: $A\uparrow$ vs control – target class	2-way ANOVA	1584	(3, 392)	F=19.86	$p < 0.001$	η_p^2=0.13	Table Supp.2
E3: $A\uparrow$ vs control – target class x ϵ	2-way ANOVA	1584	(9,1176)	F=2.55	$p < 0.01$	η_p^2=0.02	Table Supp.12
E4: $A\uparrow$ vs $A'\uparrow$– ϵ	2-way ANOVA	1556	(3, 1155)	F=17.48	$p < 0.001$	η_p^2=0.04	Table Supp.25
E4: $A\uparrow$ vs $A'\uparrow$– target class	2-way ANOVA	1556	(3, 385)	F=16.84	$p < 0.001$	η_p^2=0.12	Table Supp.2
E4: $A\uparrow$ vs $A'\uparrow$– target class x ϵ	2-way ANOVA	1556	(9,1155)	F=4.25	$p < 0.001$	η_p^2=0.03	Table Supp.12
E4: $A\uparrow$ vs $A'\uparrow$– ϵ =2, Bonferonni correction for 4 comparisons	Two-tailed t test	389	388	t=3.45	$p < 0.001$	Cohen's d=0.18	Table Supp.25
E5: self-attention vs convolution	Two-tailed t test	396	395	t=18.25	$p < 0.001$	Cohen's d=0.92	Table Supp.12
E5: Edge strength(conv) >Edge strength(self-attention)	Two-tailed t test	415	414	t=9.8	$p < 0.001$	Cohen's d = 0.68	[0.01, 0.02]

Table Supp.2: **Above-chance preference for adversarial image, by experiment and class**

Experiment	Target class(es)	Perceptual bias	test-statistic	p value	effect size (η_p^2)	95% CI of bias
2	dog	0.54	$F(1,99) = 38.9$	$< .001$	0.21	[0.03, 0.05]
	cat	0.57	$F(1,99) = 88.4$	$< .001$	0.39	[0.06, 0.09]
	bottle	0.52	$F(1,98) = 7.61$	.007	0.06	[0.0, 0.03]
	bird	0.53	$F(1,89) = 33.2$	$< .001$	0.14	[0.02, 0.05]
3	dog	0.52	$F(1,99) = 10.6$	.001	0.08	[0.01, 0.04]
	cat	0.62	$F(1,95) = 98.3$	$< .001$	0.50	[0.1, 0.15]
	bottle	0.55	$F(1,98) = 23.9$	$< .001$	0.19	[0.03, 0.07]
	bird	0.54	$F(1,100) = 18.6$	$< .001$	0.09	[0.02, 0.06]
4	cat-truck	0.54	$F(1,97) = 28.5$	$< .001$	0.17	[0.03, 0.06]
	sheep-chair	0.51	$F(1,97) = 6.85$	.010	0.07	[0.0, 0.02]
	dog-bottle	0.53	$F(1,98) = 24.6$	$< .001$	0.39	[0.02, 0.04]
	elephant-clock	0.58	$F(1,93) = 70.6$	$< .001$	0.38	[0.06, 0.1]

Table Supp.3: **Effect of ϵ on preference for adversarial image, by experiment and class (ANOVA)**

Experiment	Target class(es)	test-statistic	p value	effect size (η_p^2)
2	dog	$F(3,297) = 2.13$	.096	0.02
	cat	$F(3,297) = 10.5$	$< .001$	0.1
	bottle	$F(3,294) = 1.93$	.125	0.02
	bird	$F(3,267) = .692$	.557	0.01
3	dog	$F(3,297) = 2.53$	.057	0.03
	cat	$F(3,285) = 20.7$	$< .001$	0.18
	bottle	$F(3,294) = 5.10$	.002	0.05
	bird	$F(3,300) = 1.95$	.121	0.02
4	cat-truck	$F(3,291) = 5.08$	.002	0.05
	sheep-chair	$F(3,291) = .69$	.561	0.01
	dog-bottle	$F(3,294) = 2.53$	.058	0.03
	elephant-clock	$F(3,279) = 21.2$	$< .001$	0.19

Table Supp.4: **Effect of ϵ on preference for adversarial image, by experiment and class (Spearman Correlation between human perceptual bias and ϵ)**

Experiment	Target class(es)	ρ	p value	95% CI of ρ
2	dog	0.127	0.006	[0.03, 0.22]
	cat	0.233	< 0.001	[0.13, 0.32]
	bottle	0.104	0.019	[0.01, 0.2]
	bird	0.028	0.300	[-0.08, 0.13]
3	dog	0.105	0.018	[0.01, 0.2]
	cat	0.274	< 0.001	[0.18, 0.36]
	bottle	0.135	0.003	[0.04, 0.23]
	bird	0.062	0.110	[-0.04, 0.16]
4	cat-truck	0.150	0.001	[0.05, 0.25]
	sheep-chair	0.015	0.384	[-0.08, 0.11]
	dog-bottle	0.063	0.107	[-0.04, 0.16]
	elephant-clock	0.307	< 0.001	[0.21, 0.4]

Table Supp.5: **Variance of perceptual bias within ϵ and target class in Experiment 4**

ANOVA factor	Factor level	σ^2
Target class	cat-truck	0.014
	sheep-chair	0.011
	dog-bottle	0.010
	elephant-clock	0.019
Perturbation magnitude	$\epsilon = 2$	0.010
	$\epsilon = 4$	0.014
	$\epsilon = 8$	0.015
	$\epsilon = 16$	0.017

Table Supp.6: **Shapiro-Wilk test for normality of per-image responses - Experiment 4**

Target class	Perturbation magnitude	W	p value
cat-truck	$\epsilon = 2$	0.993	0.484
	$\epsilon = 4$	0.986	0.050
	$\epsilon = 8$	0.992	0.395
	$\epsilon = 16$	0.989	0.165
sheep-chair	$\epsilon = 2$	0.993	0.435
	$\epsilon = 4$	0.988	0.096
	$\epsilon = 8$	0.993	0.533
	$\epsilon = 16$	0.990	0.172
dog-bottle	$\epsilon = 2$	0.992	0.396
	$\epsilon = 4$	0.989	0.138
	$\epsilon = 8$	0.993	0.480
	$\epsilon = 16$	0.983	0.020
elephant-clock	$\epsilon = 2$	0.991	0.280
	$\epsilon = 4$	0.989	0.127
	$\epsilon = 8$	0.991	0.283
	$\epsilon = 16$	0.984	0.027

Table Supp.7: **Significance of the effect of a factor with shuffled labels (Experiments 1-4)**

Experiment name	Factor shuffled	# of shuffles	p value Mean ± SD
Experiment 1	(control vs $A\uparrow$)	n=10000	0.503 ± 0.292
Experiment 2	ϵ	n=10000	0.5 ± 0.288
Experiment 3	ϵ	n=10000	0.5 ± 0.289
Experiment 4	ϵ	n=10000	0.496 ± 0.29

Table Supp.8: **Wilcoxon test of difference between the test statistic (T for Experiment 1 or $F-$score for Experiments 2-4) of factor-shuffled (see Table Supp.7) and unshuffled data**

Experiment name	# of shuffled runs	Wilcoxon statistic	p value
Experiment 1	n=10000	0.0	$p < 0.001$
Experiment 2	n=10000	0.0	$p < 0.001$
Experiment 3	n=10000	0.0	$p < 0.001$
Experiment 4	n=10000	0.0	$p < 0.001$

Table Supp.9: **Wilcoxon test (one-sided) of significance of the main effect for Experiments 1-5**

Experiment name	Wilcoxon statistic	p-value	RBC (effect size)	95% CI of bias
Experiment 1	647	$p < 0.001$	0.746	$[0.052, \infty]$
Experiment 2	60302	$p < 0.001$	0.598	$[0.031, \infty]$
Experiment 3	60754	$p < 0.001$	0.546	$[0.045, \infty]$
Experiment 4	58526	$p < 0.001$	0.543	$[0.026, \infty]$
Experiment 5	65221	$p < 0.001$	0.821	$[0.103, \infty]$

Table Supp.10: **Confidence interval of perceptual bias for Experiment 4 after filtering out images with even weak evidence of class-changing perturbations (See Supplementary Note 3)**

ANOVA factor	Factor level	95% CI
Target class	cat-truck	[0.53, 0.56]
	sheep-chair	[0.5, 0.52]
	dog-bottle	[0.52, 0.54]
	elephant-clock	[0.56, 0.6]
Perturbation magnitude	$\epsilon = 2$	[0.51, 0.53]
	$\epsilon = 4$	[0.51, 0.54]
	$\epsilon = 8$	[0.54, 0.56]
	$\epsilon = 16$	[0.55, 0.58]

Table Supp.11: **Variance of perceptual bias, Experiments 1–5**

Experiment name	Category	Condition / Epsilon	Variance
Exp1	category	spider-snake	0.024
Exp1	category	cat-dog	0.016
Exp1	category	broccoli-cabbage	0.022
Exp1	condition	$A \uparrow$	0.008
Exp1	condition	control	0.011
Exp2	category	dog	0.013
Exp2	category	cat	0.016
Exp2	category	bottle	0.012
Exp2	category	bird	0.011
Exp2	epsilon	2	0.012
Exp2	epsilon	4	0.013
Exp2	epsilon	8	0.013
Exp2	epsilon	16	0.014
Exp3	category	dog	0.015
Exp3	category	cat	0.026
Exp3	category	bottle	0.021
Exp3	category	bird	0.018
Exp3	epsilon	2	0.013
Exp3	epsilon	4	0.02
Exp3	epsilon	8	0.023
Exp3	epsilon	16	0.025
Exp4	category	cat	0.014
Exp4	category	dog	0.01
Exp4	category	sheep	0.011
Exp4	category	elephant	0.019
Exp4	epsilon	2	0.01
Exp4	epsilon	4	0.014
Exp4	epsilon	8	0.015
Exp4	epsilon	16	0.017
Exp5	dog	16	0.012
Exp5	cat	16	0.017
Exp5	bottle	16	0.018
Exp5	bird	16	0.015

Table Supp.12: **95% CI of perceptual bias (Experiments 1-5)**

Experiment name	Category	Condition / Epsilon	Confidence interval
Exp1	spider-snake	$A\uparrow$	[0.26 0.32]
Exp1	spider-snake	control	[0.21 0.3]
Exp1	cat-dog	$A\uparrow$	[0.32 0.37]
Exp1	cat-dog	control	[0.24 0.31]
Exp1	broccoli-cabbage	$A\uparrow$	[0.31 0.4]
Exp1	broccoli-cabbage	control	[0.23 0.31]
Exp2	dog	2	[0.5 0.54]
Exp2	dog	4	[0.52 0.56]
Exp2	dog	8	[0.52 0.57]
Exp2	dog	16	[0.54 0.58]
Exp2	cat	2	[0.51 0.55]
Exp2	cat	4	[0.53 0.58]
Exp2	cat	8	[0.56 0.61]
Exp2	cat	16	[0.59 0.64]
Exp2	bottle	2	[0.48 0.53]
Exp2	bottle	4	[0.48 0.52]
Exp2	bottle	8	[0.5 0.54]
Exp2	bottle	16	[0.51 0.56]
Exp2	bird	2	[0.5 0.54]
Exp2	bird	4	[0.52 0.56]
Exp2	bird	8	[0.51 0.56]
Exp2	bird	16	[0.52 0.56]
Exp3	dog	2	[0.48 0.53]
Exp3	dog	4	[0.49 0.54]
Exp3	dog	8	[0.51 0.55]
Exp3	dog	16	[0.52 0.57]
Exp3	cat	2	[0.53 0.58]
Exp3	cat	4	[0.58 0.64]
Exp3	cat	8	[0.61 0.68]
Exp3	cat	16	[0.64 0.71]
Exp3	bottle	2	[0.49 0.54]
Exp3	bottle	4	[0.52 0.57]
Exp3	bottle	8	[0.52 0.58]
Exp3	bottle	16	[0.55 0.61]
Exp3	bird	2	[0.5 0.54]
Exp3	bird	4	[0.51 0.57]
Exp3	bird	8	[0.53 0.59]
Exp3	bird	16	[0.51 0.57]
Exp4	cat	2	[0.49 0.54]
Exp4	cat	4	[0.51 0.56]
Exp4	cat	8	[0.52 0.57]
Exp4	cat	16	[0.55 0.59]
Exp4	dog	2	[0.5 0.54]
Exp4	dog	4	[0.49 0.53]
Exp4	dog	8	[0.52 0.56]
Exp4	dog	16	[0.51 0.56]
Exp4	sheep	2	[0.5 0.54]
Exp4	sheep	4	[0.48 0.52]
Exp4	sheep	8	[0.49 0.53]
Exp4	sheep	16	[0.5 0.55]
Exp4	elephant	2	[0.5 0.54]
Exp4	elephant	4	[0.54 0.59]
Exp4	elephant	8	[0.58 0.64]
Exp4	elephant	16	[0.61 0.66]
Exp5	dog	16	[0.57 0.61]
Exp5	cat	16	[0.62 0.67]
Exp5	bottle	16	[0.6 0.65]
Exp5	bird	16	[0.57 0.62]

Table Supp.13: **Shapiro-Wilk normality test of residuals**

Experiment name	Main effect / Factor	W	p
Experiment 1	Main effect	0.982	0.802
Experiment 1	category	0.995	0.746
Experiment 1	condition	0.995	0.755
Experiment 2	Main effect	0.985	0.001
Experiment 2	epsilon	0.998	0.06
Experiment 2	class	0.999	0.231
Experiment 3	Main effect	0.928	$p < 0.001$
Experiment 3	epsilon	0.988	$p < 0.001$
Experiment 3	class	0.991	$p < 0.001$
Experiment 4	Main effect	0.922	$p < 0.001$
Experiment 4	epsilon	0.992	$p < 0.001$
Experiment 4	class	0.995	$p < 0.001$
Experiment 5	Main effect	0.971	$p < 0.001$
Experiment 5	category	0.98	$p < 0.001$

Table Supp.14: **Shape bias convolution**

Category	shape decisions	texture decisions	other decisions	shape bias
boat	6	49	29	10.9
knife	7	41	33	14.6
airplane	5	46	33	9.80
bear	19	44	22	30.2
dog	15	40	29	27.3
keyboard	28	42	15	40.0
cat	27	33	25	45.0
elephant	21	38	26	35.6
oven	18	35	32	34.0
bird	10	44	31	18.5
car	39	33	13	54.2
truck	39	34	12	53.4
bottle	54	26	5	67.5
chair	29	29	27	50.0
clock	62	14	9	81.6
bicycle	35	32	18	52.2
Total	414	580	359	41.7

Table Supp.15: **Shape bias attention**

Category	shape decisions	texture decisions	other decisions	shape bias
boat	10	46	29	17.9
knife	15	39	29	27.8
airplane	17	46	22	27.0
bear	25	43	17	36.8
dog	15	45	24	25.0
keyboard	23	43	19	34.9
cat	25	43	17	36.8
elephant	38	29	18	56.7
oven	34	33	18	50.8
bird	12	57	16	17.4
car	41	30	14	57.8
truck	45	36	4	55.6
bottle	56	27	2	67.5
chair	41	31	13	56.9
clock	71	10	4	87.7
bicycle	46	25	14	64.8
Total	514	583	260	46.9

Table Supp.16: **ImageNet-C Top 1 accuracy for convolution model. Model accuracy on clean ImageNet validation set is** 86.3%

Noise Type	Scale 1	Scale 2	Scale 3	Scale 4	Scale 5
Gaussian noise	81.9	79.3	73.6	63.5	44.6
Shot noise	81.5	78.5	73.1	60.3	46.9
Impulse noise	80.0	77.0	73.8	64.2	48.6
Defocus blur	78.9	75.1	65.6	54.9	43.4
Glass blur	76.7	69.2	46.9	38.3	25.4
Motion blur	83.1	80.1	73.6	63.0	54.1
Zoom blur	77.0	71.9	67.1	61.3	53.8
Snow	80.1	72.0	73.5	67.2	60.8
Frost	80.0	72.6	65.7	64.2	58.9
Fog	83.2	82.5	81.2	80.0	75.5
Brightness	84.6	83.9	82.8	80.8	78.0
Contrast	83.4	82.6	80.9	74.7	60.0
Elastic transform	81.0	63.8	75.6	67.2	42.8
Pixelate	82.9	81.8	79.7	75.6	72.1
JPEG compression	80.4	78.9	77.8	73.5	67.2
Gaussian blur	82.4	76.5	67.9	58.3	38.3
Saturate	81.9	79.6	83.9	79.8	74.4
Spatter	84.0	81.5	79.1	76.7	70.6
Speckle noise	82.1	80.6	74.6	69.7	61.8
AVG	81.3	77.2	73.5	67.0	56.7
SEM	0.50	1.21	1.96	2.40	3.30

Table Supp.17: **ImageNet-C Top 1 accuracy for attention model. Model accuracy on clean ImageNet validation set is** 86.6%

Noise Type	Scale 1	Scale 2	Scale 3	Scale 4	Scale 5
Gaussian noise	83.6	81.1	75.6	64.3	42.2
Shot noise	83.3	80.4	75.1	60.4	44.9
Impulse noise	82.6	79.5	76.1	65.0	46.4
Defocus blur	81.7	79.1	71.6	62.9	53.2
Glass blur	80.9	76.5	60.1	53.5	42.0
Motion blur	84.3	82.5	78.4	71.2	64.8
Zoom blur	79.3	74.9	70.8	66.2	59.3
Snow	81.9	75.7	77.1	72.4	67.6
Frost	81.7	75.7	69.4	68.0	62.9
Fog	83.7	82.8	81.6	80.6	76.5
Brightness	85.5	84.9	83.9	82.5	80.1
Contrast	84.3	83.4	81.4	74.0	52.4
Elastic transform	82.7	67.5	80.3	74.7	53.5
Pixelate	84.4	83.8	82.7	80.3	78.1
JPEG compression	82.3	81.2	80.2	76.8	70.4
Gaussian blur	84.1	80.7	75.1	67.8	51.1
Saturate	83.5	81.3	84.9	81.5	77.2
Spatter	85.0	83.2	81.4	81.1	77.5
Speckle noise	83.8	82.6	77.3	72.5	64.3
AVG	83.1	79.8	77.0	71.4	61.3
SEM	4.12	4.00	3.92	3.80	4.00

Table Supp.18: **Number of participants and trials per experiment.**

Experiment conditions	Number of participants	Number of trials
Experiment 2 - CAT	100	96
Experiment 2 - DOG	100	96
Experiment 2 - BIRD	90	96
Experiment 2 - BOTTLE	99	96
Experiment 3 - CAT	96	96
Experiment 3 - DOG	100	96
Experiment 3 - BIRD	101	96
Experiment 3 - BOTTLE	99	96
Experiment 4 - Sheep vs Chair	98	96
Experiment 4 - Dog vs Bottle	99	96
Experiment 4 - Cat vs Truck	98	96
Experiment 4 - Elephant vs Clock	94	96
Experiment 5 - CAT	100	96
Experiment 5 - DOG	98	96
Experiment 5 - BIRD	100	96
Experiment 5 - BOTTLE	98	96

Table Supp.19: **Accuracy of models used in Experiment 1 on ImageNet validation set.** * models trained on ImageNet with retina layer pre-pended and with train data augmented with rescaled images in the range of $[40, 255-40]$; ** model trained with adversarial examples augmented data. First ten models are models used in the adversarial training ensemble. Last two models are models used to test the transferability of adversarial examples.

**Model**	**Top-1 accuracy**
Resnet V2 101	77.0
Resnet V2 101*	72.1
Inception V4	80.2
Inception V4*	75.2
Inception Resnet V2	80.4
Inception Resnet V2*	76.6
Inception V3	78.0
Inception V3*	74.5
Resnet V2 152	77.8
Resnet V2 50*	70.8
Resnet V2 50 (test)	75.6
Inception V3** (test)	77.6

Table Supp.20: **Top-1 classification accuracy (%) of models used in Experiments 2 − 4 on ImageNet validation set**

Model name	Top-1 accuracy
EfficientNet-B4	83
EfficientNet-B5	83.7
ResNet-101	79.8
InceptionV4	80
PNASNet-5	82.9
HaloNet	80.6

Table Supp.21: **Top-1 classification accuracy (%) of models used in Experiment 5 on ImageNet validation set**

Model name	Top-1 accuracy
ViT-B/32	80.7
ResNet-101x1	80.7
ViT-L/16	87.1
ResNet-200x3	87.2

Table Supp.22: **Calibration temperature of ImageNet-trained models**

Model name	Calibration temperature
EfficientNet-B4	0.782
EfficientNet-B5	0.835
ResNet-101	0.967
InceptionV4	0.937
PNASNet-5	0.867
HaloNet	0.825

Table Supp.23: **Average per-trial perceptual bias of EfficientNetB2 (blackbox ANN)**

Experiment identifier	$\epsilon = 2$	$\epsilon = 4$	$\epsilon = 8$	$\epsilon = 16$
E2	1.0 ± 0.00	1.0 ± 0.00	1.0 ± 0.00	1.0 ± 0.00
E3	0.95 ± 0.01	0.99 ± 0.01	1.0 ± 0.00	1.0 ± 0.00
E4	0.85 ± 0.01	0.97 ± 0.01	1.0 ± 0.00	1.0 ± 0.00

Table Supp.24: **Number of ImageNet fine classes (ImageNet support) corresponding to each of our experiment's coarse categories**

Coarse category	Number of ImageNet fine categories
Cat	6
Dog	120
Bird	52
Bottle	7
Sheep	6
Chair	4
Elephant	2
Clock	3
Truck	8

Table Supp.25: **Experiments 2–4 - 95% CI of perceptual bias by ϵ**

**Experiment**	**Perturbation magnitude**	**95% CI of perceptual bias**
Experiment 2	$\epsilon = 2$	[0.51, 0.53]
	$\epsilon = 4$	[0.52, 0.54]
	$\epsilon = 8$	[0.53, 0.56]
	$\epsilon = 16$	[0.55, 0.57]
Experiment 3	$\epsilon = 2$	[0.51, 0.53]
	$\epsilon = 4$	[0.54, 0.56]
	$\epsilon = 8$	[0.55, 0.58]
	$\epsilon = 16$	[0.57, 0.60]
Experiment 4	$\epsilon = 2$	[0.51, 0.53]
	$\epsilon = 4$	[0.51, 0.54]
	$\epsilon = 8$	[0.54, 0.56]
	$\epsilon = 16$	[0.55, 0.58]

G Supplementary Figures

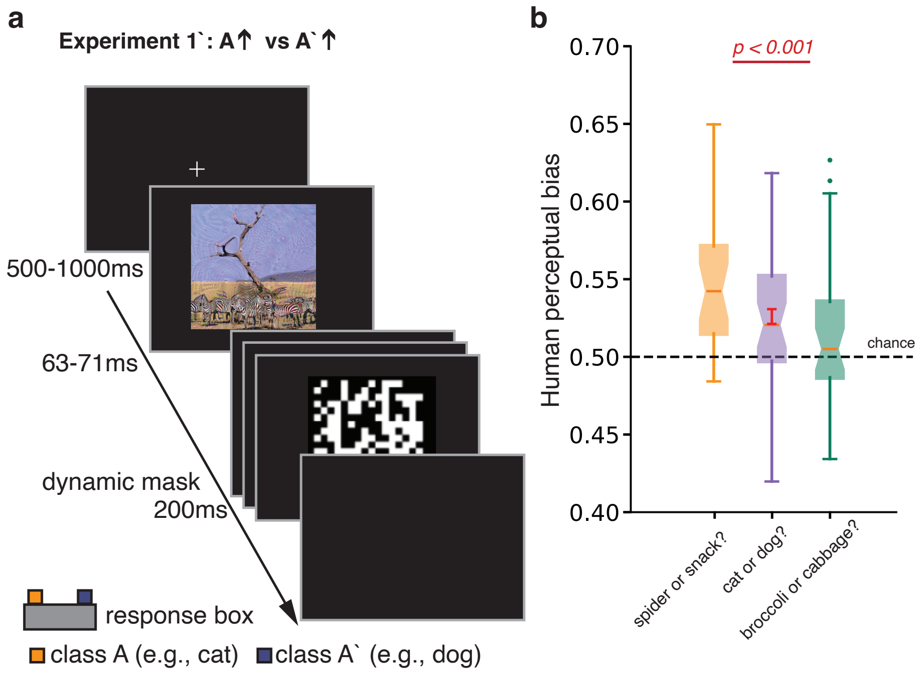

Figure Supp.1: **Experiment SI-1: Adversarial examples bias the perception of time-limited humans (a)** Task structure: participants chose between class A and class A' when briefly presented with image stimuli followed by high contrast mask. Images used in the experiment are obtained from ImageNet dataset [61], but image used here as an illustration is obtained from MS-COCO dataset [62]. (b) Human perceptual bias. Red error bar show (mean ± S.E. across participants and classes) of adversarial examples increasing the confidence of n=38 independent participants (spider vs snack n=24, cat vs dog n=35, broccoli vs cabbage n=32) in target classes ($t(37) = 6.016, p < 0.001$, Cohen's d=0.97, 95% CI of bias=[0.02, 0.04], two-tailed ttest); colored box-and-whisker plots show the perceptual bias distribution from human participants for each of the image class pairs (same format as Figure 2c).

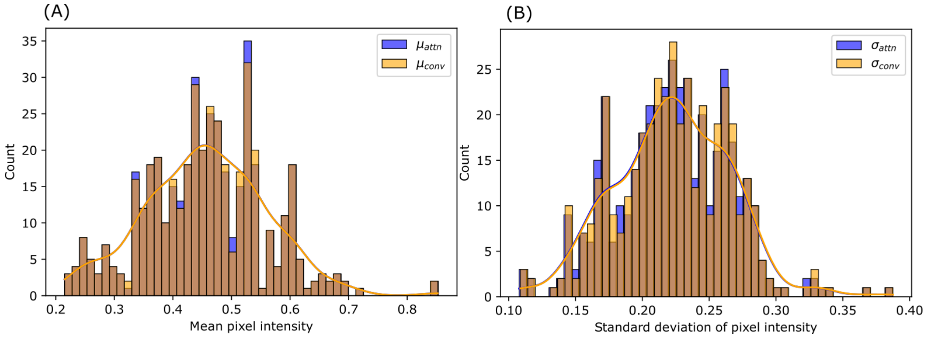

Figure Supp.2: **Comparing low-level image statistics of self-attention and convolution adversarial images.** We compared the distribution of the mean pixel intensity (A) and standard deviation of the pixel intensity (B) of adversarial images created using the self-attention and convolutional ANN. We could not find a significant difference between the mean pixel intensity distribution of self-attention and convolutional adversarial images (Kolmogorov Smirnov test, $Z = 0.007$, $p = 0.99$, effect size $D = 0.0005$). Likewise, we could not find significant difference between their standard deviation distributions (Kolmogorov Smirnov test, $Z = 0.019, p = 0.99$, effect size $D = 0.001$). These results suggest that our observed human perceptual effects cannot be explained by low-level image statistics related to luminance and contrast.

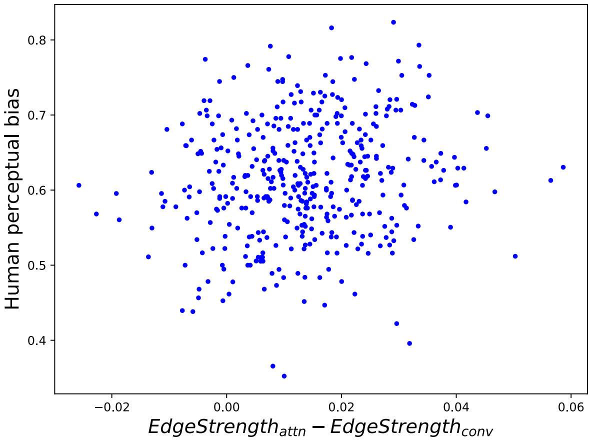

Figure Supp.3: **Effect of perturbation edge strength** We computed edge strength of perturbation images corresponding to self-attention and convolutional ANNs respectively as the number of unit length edges obtained by processing perturbation images with Canny edge detection. We observe that the greater the difference in edge strength between a pair of attention and convolutional ANN perturbations, the more likely are human participants to choose the perturbation corresponding to the former. Human preference for one image in a pair is positively correlated with the difference in edge strength of the perturbations forming the pair (Spearman's $\rho = 0.158$, $p = 0.001$, 95% CI of ρ=[0.06, 0.25]).

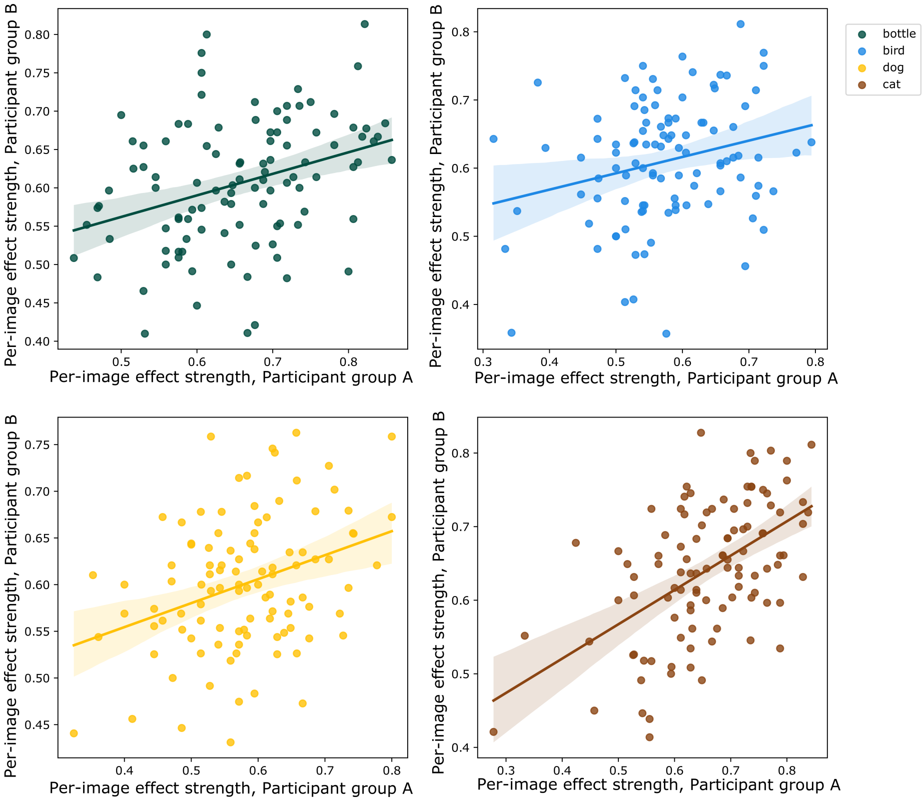

Figure Supp.4: **Individual differences in stimulus effectiveness** For each of Experiment 5's HITs, we studied the correlation between the perceptual bias of each image pair on two non-overlapping equally-sized pool of our participants. We show the regression line for predicting Participant group B's per-image effect strength from Participant group A's per-image effect strength and the 95% confidence interval corresponding to that fit. We observed that there exists a strong correlation (E5, Bottle: Pearson's $\rho = 0.497, p < 0.001$, 95% CI of ρ=[0.337, 0.63], Bird: Pearson's $\rho = 0.388, p < 0.001$, 95% CI of ρ=[0.211, 0.540], Dog: Pearson's $\rho = 0.262, p = 0.007$, 95% CI of ρ=[0.074, 0.433] Cat: Pearson's $\rho = 0.503, p < 0.001$, 95% CI of ρ=[0.344, 0.634]) suggesting the presence of adversarial images that affect humans collectively with varied degrees of effectiveness.

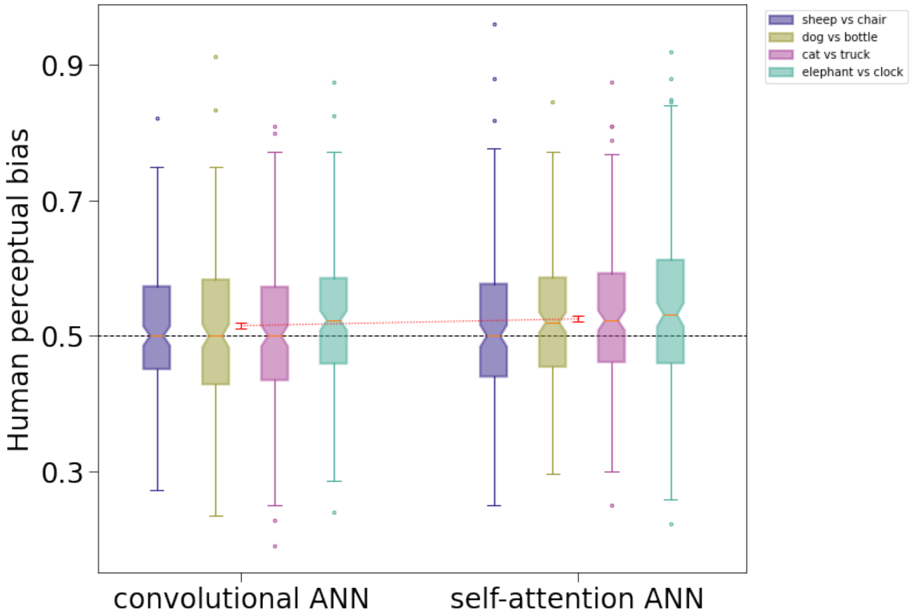

Figure Supp.5: **Experiment SI-2: Both convolutional and self-attention ANNs yield effective adversarial images that bias participants.** We conducted a version of Experiment 4 with $\epsilon \in \{4, 8\}$ in which one half of the experiment's adversarial image pairs were produced by convolutional net and the other half of the experiment's image pairs were produced by a self-attention net. The figure shows human perceptual bias for n=381 independent participants (sheep vs chair n=90, dog vs bottle n=98, cat vs truck n=98, elephant vs clock n=95) for each of the four target class pairs used in the Experiment, collapsed over ϵ values (bar and boxplots use same convention as Figure 2c). Both the convolutional and self-attention ANNs produce adversarial images that reliably bias participants; averaging across target classes and ϵ, the bias (deviation from chance) for convolutional ANNs is 1.51% ($t(380) = 3.91, p < 0.001$, Cohen's d=0.2, 95% CI of bias = [0.01, 0.02], two-sided ttest) and for self-attention ANNs in 2.52% ($t(380) = 5.98, p < 0.001$, Cohen's d=0.31, 95% CI of bias = [0.02, 0.03], two-sided ttest) , but we do not find credible evidence that these biases are different (paired $t(379) = 1.87, p = 0.062$, Cohen's d=0.13, 95% CI of difference between means = [0.0, 0.2], two-tailed ttest). We also conducted a three-way ANOVA with the factors: class pair, ϵ, and model (convolutional vs. self-attention). Other than the overall bias being reliably nonzero ($F(1, 377) = 45.2, p < 0.001, \eta_p^2 = 0.107$, 95% CI of mean bias=[0.01, 0.03]), all other effects and interactions were non-significant, including main effects of ϵ ($F(1, 377) = 3.61, p = 0.058, \eta_p^2 = 0.009$) and target classes ($F(3, 377) = 2.43, p = .065, \eta_p^2 = 0.019$). We therefore consider this experiment inconclusive in determining the relative effectiveness of convolutional and self-attention models.

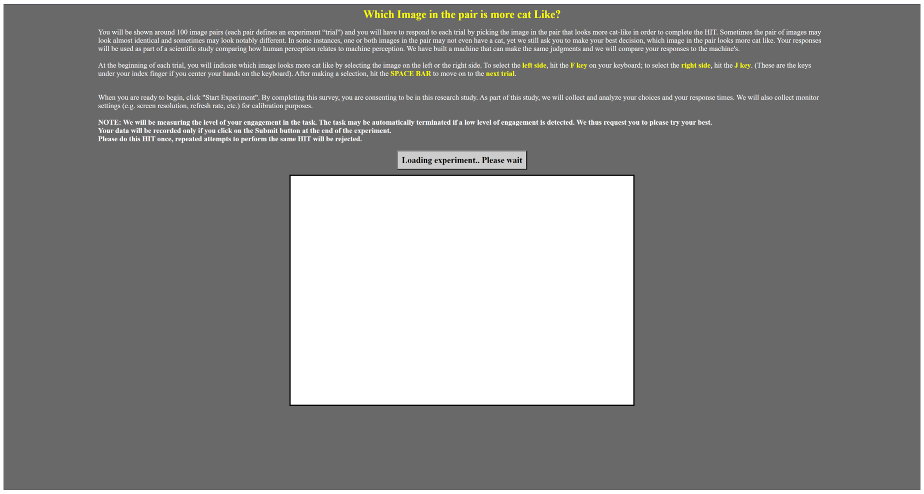

Figure Supp.6: **Experiment 2 introduction screenshot for target class “CAT”.** Web applications that the screenshots are taken from was written using HTML, JavaScript and jQuery with helper code from Zhou & Firestone, 2019[33].

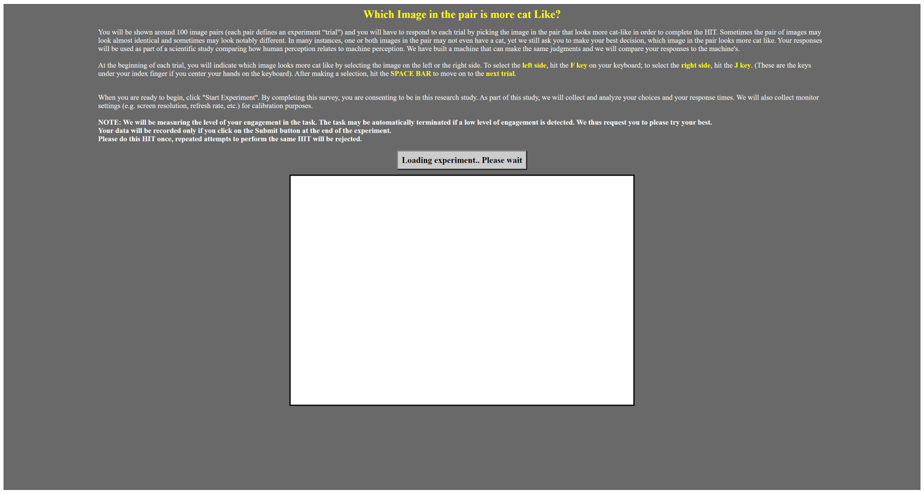

Figure Supp.7: **Experiment 3 introduction screenshot for target class “CAT”.** Web applications that the screenshots are taken from was written using HTML, JavaScript and jQuery with helper code from Zhou & Firestone, 2019[33].

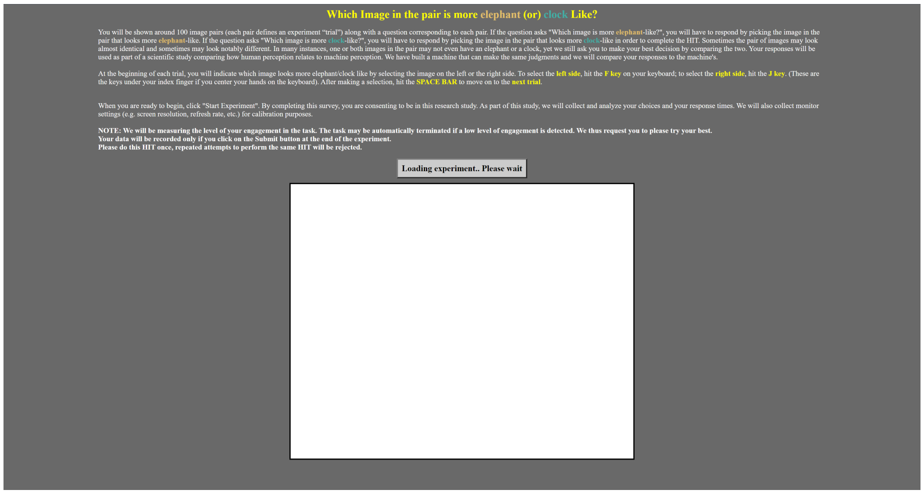

Figure Supp.8: **Experiment 4 introduction screenshot for target classes “elephant vs clock”.** Web applications that the screenshots are taken from was written using HTML, JavaScript and jQuery with helper code from Zhou & Firestone, 2019[33].

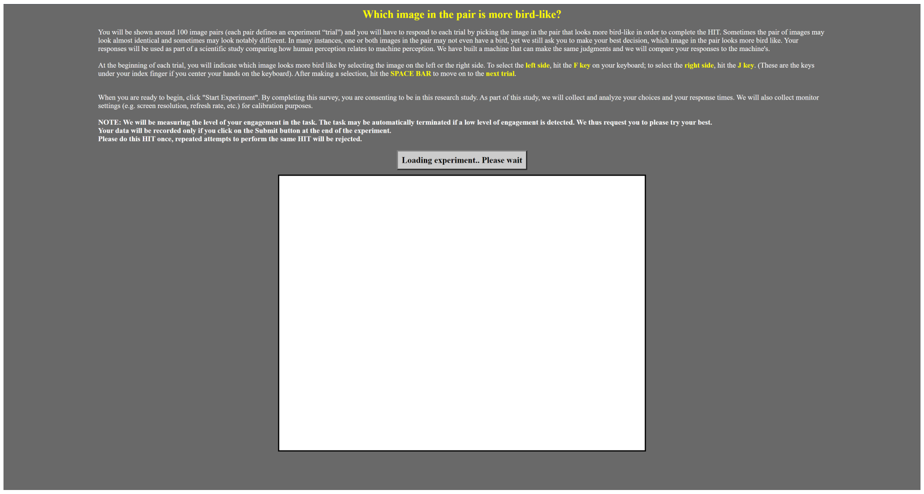

Figure Supp.9: **Experiment 5 introduction screenshot for target class “BOTTLE”.** Web applications that the screenshots are taken from was written using HTML, JavaScript and jQuery with helper code from Zhou & Firestone, 2019[33].

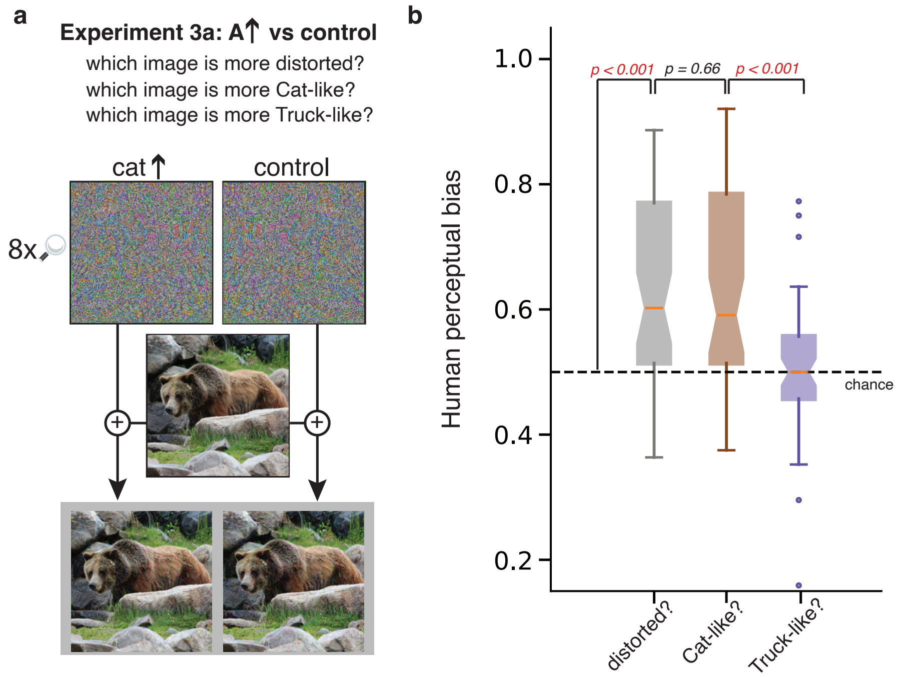

Figure Supp.10: **Experiment SI-3** (a) This panel describes a control experiment in which participants are shown the same stimuli as the $\epsilon = 16$ 'cat' condition of Experiment 3. Each trial consists of a pair of images, one adversarially perturbed toward the cat class and one with the same perturbation only flipped left-right. In a replication condition, we ask participants which image is more cat-like. In a second condition, we ask which image is more distorted. And in a third condition we ask, which image is more like a truck (i.e., a class which is different from the adversarial class). The three conditions were between participant, with n=50 independent participants per condition. Example image is drawn from a collection of images from the Microsoft COCO dataset[62] and OpenImages dataset[63]. (b) Box plots (same convention as Figure 2c) showing that both the 'distorted' condition and the 'cat' condition obtained a reliable bias (from n=50 independent participants) in selecting the adversarial image (distorted: $t(49) = 6.241, p < 0.001$, Cohen's d=0.88, 95% CI of bias=[0.09, 0.17], two-tailed ; cat: $t(49) = 6.33, p < 0.001$, Cohen's d=0.9, 95% CI of bias=[0.1, 0.18], two-tailed ttest). However, there was no reliable difference between the two conditions ($t(49) = 0.439, p = 0.66$, Cohen's d=0.09, 95% CI of bias=[-0.05, 0.07], two-tailed). However, we find no credible evidence that the 'truck' condition produced any deviation from chance ($t(49) = 0.545, p = 0.588$, Cohen's d=0.077, 95% CI of bias=[-0.02, 0.04], two-tailed), and the 'cat' bias was larger than the 'truck' bias ($t(49) = 4.984, p < 0.001$, Cohen's d=0.996, 95% CI of the difference between their means is computed as greater than 0.09, one-tailed ttest). Thus, the experiment shows that choice based on image distortion could produce a bias as large as we observed for the $\epsilon = 16$ 'cat' condition of Experiment 3, in actuality participants were not choosing based on distortion. Were participants choosing based on distortion, the adversarial class would be irrelevant, and one would expect no difference between the 'cat' and 'truck' conditions, which we observe here.

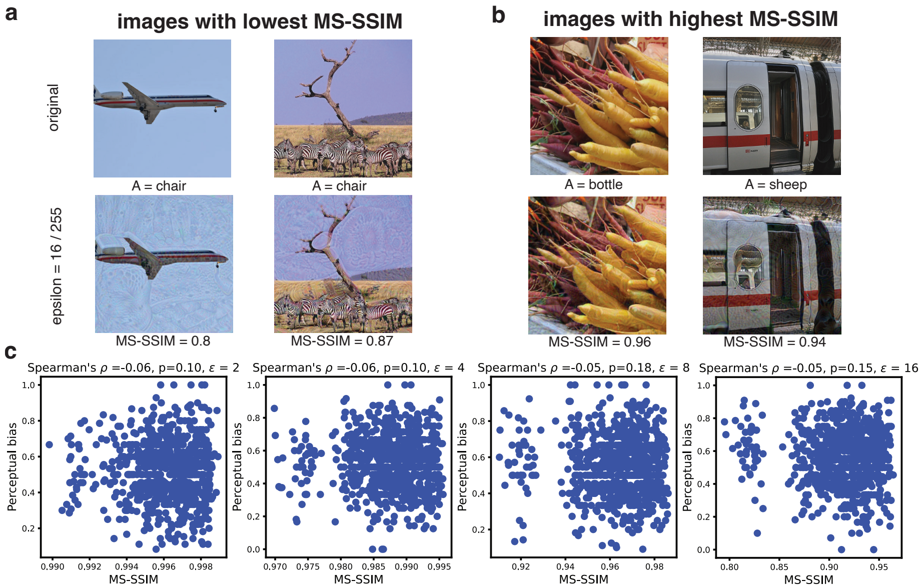

Figure Supp.11: **MS-SSIM and perceptual bias**. In this Figure, we examine the relationship between MS-SSIM and the perceptual bias induced by an adversarial image (i.e., the probability above chance that participants chose the adversarial image). MS-SSIM, the multi-scale structural similarity index [36], measures the perceptual similarity of an original image and perturbed image. The images with the lowest and highest MS-SSIM indices are shown in (a) and (b), respectively. For a given magnitude of perturbations, MS-SSIM is a measure of saliency of the perturbations and is also a good proxy for homogeneity of image backgrounds. Images in a and b are obtained from the Microsoft COCO dataset[62] and OpenImages dataset[63]. In (c), we show scatterplots of MS-SSIM versus perceptual bias at different magnitudes of adversarial perturbations (ϵ). For each scatter plot, we use the same set of the original images. For each perturbation magnitude, we find no credible evidence that participants are more sensitive to perturbations that are highly salient (e.g., textures painted into a uniform background such as the sky) than ones that are less salient, as measured by MS-SSIM (Spearman's rank correlation at $\epsilon = 2 : \rho = -0.06$, p = 0.10, 95% CI of ρ=[-0.13, 0.01]; $\epsilon = 4 : \rho = -0.06$, p = 0.10, 95% CI of ρ=[-0.13, 0.01]; $\epsilon = 8 : \rho = -0.05$, p = 0.18, 95% CI of ρ=[-0.12, 0.02]; $\epsilon = 16 : \rho = -0.05$, p = 0.15, 95% CI of ρ=[-0.12, 0.02]). Note also that MS-SSIM range decreases across the different values of ϵ simply reflecting the fact that images have larger perturbation as ϵ increases not that homogeneity of images change.

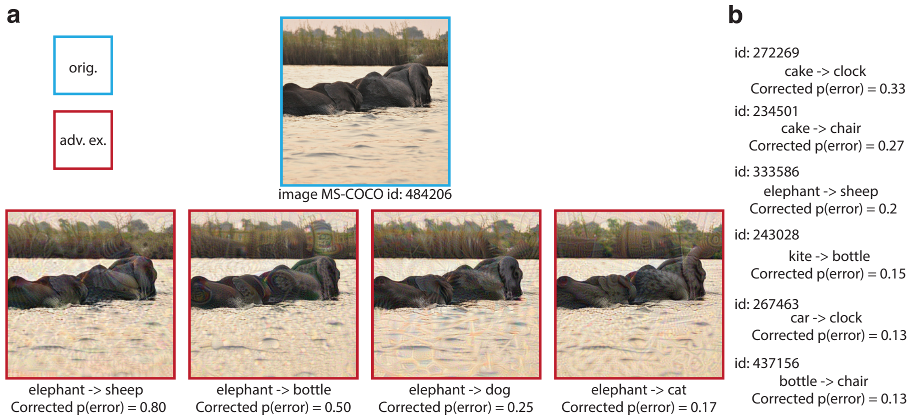

Figure Supp.12: **Experiment SI-4.** In this experiment, participants chose a response class from 10 alternatives, one of which was always the true image class (T), one was the adversarial class (A), and one was an alternative class (A'). This experiment included pairs of perturbed images that came from the same source image, one warped from target class T to adversarial class A ($T \to A$) and one to a different class A' ($T \to A'$), all with $\epsilon = 16$. The Figure lists the 10 $T \to A$ images with the highest corrected misclassification rate along with the corresponding original image (probability of classification error for each image is shown below each image). Image examples shown in this figure are from the publicly available MS-COCO[62] and OpenImages[63] datasets. (a) shows the image that is got conssistency misclassified in multimple setting. (b) Lists MS-COCO[62] ids for the rest of the top-10 images with highest classifications.

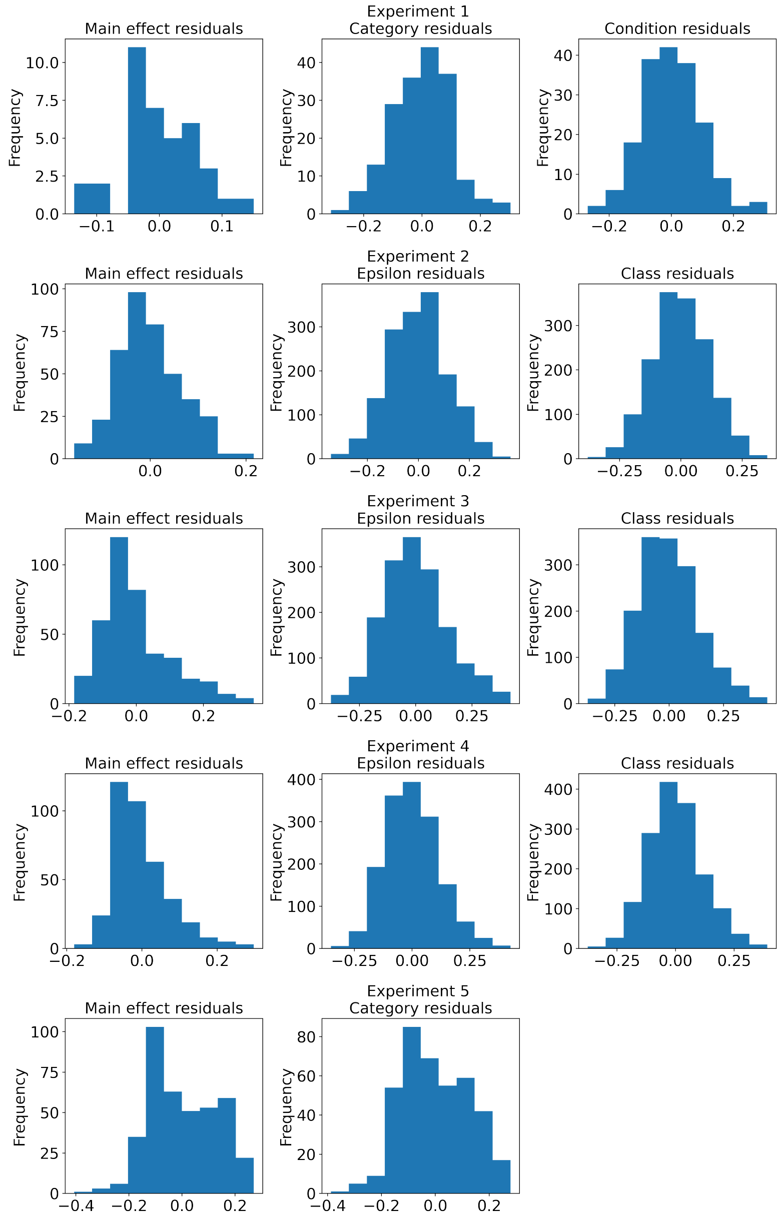

Figure Supp.13: **Residuals of the perceptual bias for Experiments 1-5.** We computed residuals corresponding to the main effect and the effect of independent factors for each experiment, these residuals are shown in the above plot. We note that the distribution of residuals in each case is unimodal. We report statistics for Shapiro-Wilk test of normality of each of the above residuals in Table Supp.13.
