# Supplementary material for: Subtle adversarial image manipulations influence both human and machine perception
Source: Nat Commun. 2023 Aug 15;14:4933. doi: 10.1038/s41467-023-40499-0 (PMC10427626; doi:10.1038/s41467-023-40499-0)
Supplement: Supplementary file 2 — Reporting Summary [file 41467_2023_40499_MOESM2_ESM.pdf]

nature portfolio

Corresponding author(s): Gamaleldin F. Elsayed

Last updated by author(s): 07/20/2023

Reporting Summary

Nature Portfolio wishes to improve the reproducibility of the work that we publish. This form provides structure for consistency and transparency in reporting. For further information on Nature Portfolio policies, see our Editorial Policies and the Editorial Policy Checklist.

Statistics

For all statistical analyses, confirm that the following items are present in the figure legend, table legend, main text, or Methods section.

n/a	Confirmed	
☐	☒	The exact sample size (*n*) for each experimental group/condition, given as a discrete number and unit of measurement
☐	☒	A statement on whether measurements were taken from distinct samples or whether the same sample was measured repeatedly
☐	☒	The statistical test(s) used AND whether they are one- or two-sided<br>*Only common tests should be described solely by name; describe more complex techniques in the Methods section.*
☐	☒	A description of all covariates tested
☐	☒	A description of any assumptions or corrections, such as tests of normality and adjustment for multiple comparisons
☐	☒	A full description of the statistical parameters including central tendency (e.g. means) or other basic estimates (e.g. regression coefficient) AND variation (e.g. standard deviation) or associated estimates of uncertainty (e.g. confidence intervals)
☐	☒	For null hypothesis testing, the test statistic (e.g. *F*, *t*, *r*) with confidence intervals, effect sizes, degrees of freedom and *P* value noted<br>*Give P values as exact values whenever suitable.*
☒	☐	For Bayesian analysis, information on the choice of priors and Markov chain Monte Carlo settings
☐	☒	For hierarchical and complex designs, identification of the appropriate level for tests and full reporting of outcomes
☐	☒	Estimates of effect sizes (e.g. Cohen's *d*, Pearson's *r*), indicating how they were calculated

Our web collection on statistics for biologists contains articles on many of the points above.

Software and code

Policy information about availability of computer code

Data collection	We used HTML, CSS, Javascript and PsychoPy to write code that performed data collection. Code packages for generating experiments in HTML, Javascript and jQuery are available at https://osf.io/dnmkw/
Data analysis	We used Python packages NumPy, Pandas, SciPy, Scikit-Learn and Matplotlib for data analysis. Python notebooks for generating results are also included in https://osf.io/dnmkw/

For manuscripts utilizing custom algorithms or software that are central to the research but not yet described in published literature, software must be made available to editors and reviewers. We strongly encourage code deposition in a community repository (e.g. GitHub). See the Nature Portfolio guidelines for submitting code & software for further information.

Data

Policy information about availability of data

All manuscripts must include a data availability statement. This statement should provide the following information, where applicable:

- Accession codes, unique identifiers, or web links for publicly available datasets
- A description of any restrictions on data availability
- For clinical datasets or third party data, please ensure that the statement adheres to our policy

For our experiments' stimuli, we reused images from ImageNet [Deng et al, 2009], Microsoft COCO [Lin et al, 2014], and OpenImages [Kuznetsova et al, 2020]. The human participant responses generated and/or analysed during the current study are available at https://osf.io/dnmkw/. Illustration images from Figure 1 were obtained with permission from [Yuan et al, 2020; Zhou & Firestone, 2019]. Images displayed in the manuscript are licensed under the Creative Commons BY 2.0

Attribution license (https://creativecommons.org/licenses/by/2.0/).

Research involving human participants, their data, or biological material

Policy information about studies with human participants or human data. See also policy information about sex, gender (identity/presentation), and sexual orientation and race, ethnicity and racism.

Reporting on sex and gender	This study did not include data on sex and gender.
Reporting on race, ethnicity, or other socially relevant groupings	N/A
Population characteristics	We specified the pool of participants in our experiments to be those residing in North America.
Recruitment	For experiment 1 and SI 1, we advertised the need of research participants to contribute to a human perception study and subjects from the same institution (who are not involved or related to the research team) applied voluntarily. For experiments 2-5 and SI 2-5, we used Mechanical Turk -- a common online rating platform -- to conduct our experiments and subjects participated voluntarily.
Ethics oversight	We have complied with all relevant ethical regulations for our behavioral experiment procedures. Our experimental protocol was granted an Institutional Review Board (IRB) exemption by an external, independent, ethics board (Quorum review ID 33016).

Note that full information on the approval of the study protocol must also be provided in the manuscript.

Field-specific reporting

Please select the one below that is the best fit for your research. If you are not sure, read the appropriate sections before making your selection.

☐ Life sciences ☒ Behavioural & social sciences ☐ Ecological, evolutionary & environmental sciences

For a reference copy of the document with all sections, see nature.com/documents/nr-reporting-summary-flat.pdf

Behavioural & social sciences study design

All studies must disclose on these points even when the disclosure is negative.

Study description	We performed a behavioral study to quantitatively measure the susceptibility of human research participants to "adversarial images" that are created to fool artificial neural networks for image classification. Our study confines to a 2-Alternative forced choice design structure where participants responded to individual trials consisting of adversarial image stimuli. These responses were analyzed quantitatively to measure human susceptibility to adversarial images and test hypotheses concerning the cause for such susceptibility.
Research sample	We specified the pool of participants in our HITs to be located in North America.
Sampling strategy	Sample size was determined to be comparable to related previous studies and was determined prior to conducting the experiments.
Data collection	Experiment 1, SI 1: High refresh rate computer screen (ViewSonic XG2530) in a room with dimmed light. Subjects were asked to classify images that appeared on the screen to one of two classes (two alternative forced choice) by pressing buttons on a response time box (LOBES v5/6:USTC) using two fingers on their right hand. Experiments 2-5, SI 2-5: Data collection was performed on Amazon Mechanical Turk, a commonly used online rating platform through web browser. Since the data was collected remotely, we the researchers were not present besides the participants.
Timing	Experiment 1 and Experiment SI 1's data were collected in January 2018. Experiments 2-5 and SI 2 data were collected between October 2020 to June 2021. Experiments SI 3-5 were collected between January 2022 and April 2022.
Data exclusions	We placed catch trials in Experiments 2-5 and SI 2-5 (i.e., trials with clear correct answer) to measure the level of engagement in the task. If subjects failed those catch trials the task would automatically terminate and data would not be analyzed.
Non-participation	N/A
Randomization	We used a common online rating platform to conduct our experiments and subject allocation to group will be roughly random. However, we can't guarantee complete randomness as we don't have complete control over subject enrollment in our HITs.

Reporting for specific materials, systems and methods

We require information from authors about some types of materials, experimental systems and methods used in many studies. Here, indicate whether each material, system or method listed is relevant to your study. If you are not sure if a list item applies to your research, read the appropriate section before selecting a response.

Materials & experimental systems

n/a	Involved in the study
☒	☐ Antibodies
☒	☐ Eukaryotic cell lines
☒	☐ Palaeontology and archaeology
☒	☐ Animals and other organisms
☒	☐ Clinical data
☒	☐ Dual use research of concern
☒	☐ Plants

Methods

n/a	Involved in the study
☒	☐ ChIP-seq
☒	☐ Flow cytometry
☒	☐ MRI-based neuroimaging
